# A scoping review of vulvodynia research: Diagnosis, treatment, and care experiences

**DOI:** 10.1177/17455057251345946

**Published:** 2025-06-17

**Authors:** Athina Zoi Lountzi, Purva Abhyankar, Hannah Durand

**Affiliations:** 1 Division of Psychology, University of Stirling, Scotland, UK

**Keywords:** vulvodynia, vulval pain, clinical, psychosocial research

## Abstract

**Background:**

Vulvodynia is a significant genital pain condition, affecting an estimated 10% to 28% of individuals worldwide. Its multifactorial etiology, diagnostic challenges, and limited treatment options contribute to its substantial personal and socioeconomic burden. Despite its prevalence, vulvodynia remains under-recognized and under-researched, necessitating a comprehensive review of existing evidence to inform future research strategies.

**Objective:**

This scoping review examines the extent and nature of clinical and psychosocial research on vulvodynia, with a focus on diagnosis, treatment, healthcare access, and its impact on quality of life, psychological well-being, and intimate relationships.

**Eligibility Criteria:**

Eligible studies included primary research using quantitative, qualitative, or mixed methods designs, as well as systematic, scoping, and topical reviews. Studies were included if they examined clinical or psychosocial aspects of vulvodynia. Research on other types of vulvar pain, animal studies, neurobiological research, and studies from non-high-income countries were excluded.

**Sources of Evidence and Methods:**

A systematic search of Medline, PubMed, CINAHL, PsycINFO, and Cochrane was conducted in March 2024 using predefined search terms related to vulvodynia, diagnosis, treatment, and patient experiences. Review findings, limitations, and recommendations were extracted to provide an overview of existing research, mapping methodologies, measures, and key findings of primary studies on vulvodynia.

**Results:**

A total of 144 articles were included, comprising 21 reviews and 123 primary studies. Clinical research primarily addressed diagnosis, risk factors, and comorbidities, while treatment studies evaluated pharmacological therapies, psychological therapies, laser therapy, physiotherapy, acupuncture, and multidisciplinary approaches. Psychosocial research focused on patient experiences, psychosocial factors, and barriers to care. However, methodological limitations, inconsistent measurement tools, limited patient involvement, and study heterogeneity challenge the generalizability of findings.

**Conclusions:**

This review highlights critical gaps in vulvodynia research. Despite considerable research efforts, vulvodynia remains poorly understood. Addressing methodological weaknesses and involving patients more robustly in research design are essential to advance knowledge and improve care outcomes in vulvodynia.

## Plain language summary

**Understanding vulvodynia: What research tells us about diagnosis, treatment, and patient experiences** Vulvodynia is a chronic pain condition affecting the vulva that can cause burning, stinging, or sharp pain, sometimes making daily activities and sexual intercourse difficult. Despite affecting up to 1 in 10 women, vulvodynia remains poorly understood, underdiagnosed, and undertreated. Many people with vulvodynia face long delays in getting a diagnosis, inconsistent treatment recommendations, and a lack of clear care pathways. This study reviews clinical and psychosocial research on vulvodynia published between 2015 and 2024, analyzing 144 studies to understand how the condition is diagnosed, treated, and experienced. Our findings show that diagnostic criteria and treatment approaches vary widely, making it difficult for healthcare providers to offer consistent care. Treatments range from medications (e.g. lidocaine, antidepressants, gabapentin), physiotherapy, psychological therapies (cognitive behavioral therapy, acceptance and commitment therapy), laser treatment, and even surgery. However, there is no universal treatment plan, and many approaches lack strong evidence to support their effectiveness. We also found that psychosocial factors, such as anxiety, depression, and relationship difficulties, play a significant role in the impact of vulvodynia. Many women feel dismissed by healthcare professionals and experience stigma, frustration, and isolation when seeking help. Peer support and mental health care were identified as crucial but under-researched areas. Overall, this review highlights critical gaps in vulvodynia research, including the need for standardized diagnosis and treatment guidelines, better outcome measures, and greater involvement of patients in research design. Addressing these gaps can help improve awareness, treatment options, and patient experiences.

**Key Takeaway:** Vulvodynia research has grown in recent years, but many knowledge gaps remain. Improving diagnosis, treatment, and healthcare experiences is critical to supporting those living with this condition.

## Introduction

Vulvodynia is an idiopathic pain disorder of the female genital area, the vulva. According to the International Society for the Study of Vulvovaginal Disease (ISSVD), vulvodynia is defined as chronic vulvar pain persisting for a minimum of 3 months, lacking a definitive, identifiable cause, and potentially influenced by associated factors.^1^ The pain can be localized (e.g. vestibulodynia, clitorodynia) or generalized in and/or around the vulva, with symptoms including soreness, burning, and/or stinging. Pain can be elicited by contact (provoked) and/or arise spontaneously.^2^ Vulvodynia is estimated to impact between 10% and 28% of women of reproductive age, with an estimated incidence rate of 4.2%.^3,4^ It is more prevalent among younger women and those with preexisting psychological conditions and concurrent pain disorders.^3–5^ The worldwide impact of vulvodynia remains uncertain, as there is a scarcity of epidemiological studies, particularly in lower-income countries.^3^ Furthermore, the diagnosis of vulvodynia is primarily one of exclusion, requiring the elimination of other identifiable causes of vulvar pain, leading to underdiagnosis and potential misdiagnosis with similar vulvar pain disorders such as vaginismus, vaginitis, or pudendal neuralgia.^6–8^ Importantly, fewer than 50% of individuals who experience vulvar pain seek medical help due to a variety of reasons ranging from structural and societal barriers (e.g. stigmatization, discrimination) to personal barriers (e.g. psychological and interpersonal relationship factors).^9,10^

The etiology of vulvodynia is unknown and considered to be multifactorial. A variety and combination of biopsychosocial factors have been suggested to trigger the onset and duration of vulvodynia, such as inflammation, chronic infections, hormonal factors, nerve damage, genetic predisposition, musculoskeletal patterns, as well as psychological factors.^11^ Due to its complexity, treating vulvodynia presents significant challenges, and available treatment modalities demonstrate limited success rates. Currently, there are no standardized guidelines for managing vulvodynia, and treatment options vary widely, encompassing topical (e.g. lidocaine, gabapentin) and oral medications (e.g. tricyclic antidepressants), self-care strategies, physiotherapy (e.g. pelvic floor treatment, soft tissue work), psychotherapy, and surgical interventions.^12^ A multidisciplinary approach to treatment is imperative to address both the physiological and psychological dimensions of living with and managing vulvodynia^13^; however, the extent to which this occurs in practice is unclear.

The combination of low help-seeking rates and limited treatment success poses a significant concern for individuals with vulvodynia. The complexity of diagnosing vulvodynia is exacerbated by a general lack of awareness among healthcare professionals. Many women report that their healthcare professionals lack awareness and education about vulvodynia, contributing to feelings of stigma, confusion, and embarrassment following healthcare consultations.^14^ Women often report feeling unheard and dismissed by healthcare professionals, with their symptoms and pain experiences being minimized and misunderstood.^15,16^ Societal normalization of women’s pain compounds the general lack of awareness of vulvodynia as a chronic condition, often requiring persistence from individuals to secure a diagnosis. Many women remain unaware of available treatment options.^17,18^ These challenges may discourage individuals from seeking help, resulting in adverse health and well-being-related outcomes.

Vulvodynia has historically been under-recognized and under-researched. It is only in recent years that vulvodynia has been recognized as a health condition, with the term ‘idiopathic vulvar pain’ being accepted for the first time in 1976.^2^ The broad acceptance of vulvodynia’s multifactorial etiology did not materialize until 2015, marking a pivotal moment in the academic and clinical recognition of the condition’s complex origins.^2^ The recent recognition of vulvodynia, coupled with its complex etiology and the lack of awareness and educational resources concerning vulvodynia, highlights the need for enhanced research attention. Mapping the existing clinical and psychosocial literature on vulvodynia to provide a broad overview of current evidence can help identify gaps to guide future research. Thus, this review aimed to identify the nature and extent of clinical and psychosocial research on vulvodynia, focusing on diagnosis, treatment, healthcare access, and its impact on quality of life, physical and psychological well-being, and intimate relationships across settings.

## Methods

This scoping review was conducted in accordance with the JBI methodology for scoping reviews. Searches of PROSPERO, MEDLINE, the Cochrane Database of Systematic Reviews, and *JBI Evidence Synthesis* were conducted and no current or in-progress scoping reviews or systematic reviews on the topic were identified. The Preferred Reporting Items for Systematic reviews and Meta-Analyses extension for Scoping Reviews (PRISMA-ScR) checklist^19^ was used to guide the reporting of this review (see Supplemental Appendix A). A protocol was developed prior to conducting the review and is publicly available on the Open Science Framework (https://osf.io/gyf8x/).

### Review questions

This scoping review aimed to address the following research questions:

1. What clinical and/or psychosocial research exists on diagnosis and treatment of vulvodynia?
2. What clinical and/or psychosocial research exists on patient experience of vulvodynia?
3. What clinical and/or psychosocial research exists on seeking/accessing/receiving care for vulvodynia?

### Inclusion criteria

#### Participants

This review considered studies that included individuals diagnosed by a healthcare professional with any type of vulvodynia, including localized (e.g. vestibulodynia, clitorodynia), generalized, provoked, spontaneous, or mixed, with any temporal pattern, and any onset. Considering the aims of this review, studies that included healthcare professionals with experience in vulvodynia treatment as participants were also considered.

#### Concept

This review considered studies describing clinical and psychosocial research on vulvodynia, focusing on three key areas: (1) diagnosis and treatment; (2) patient experience, including quality of life, physical and psychosocial well-being, and impact on relationships; and (3) healthcare access, encompassing seeking, accessing, and receiving care. Studies on other types of vulval pain, vulval pain with an identifiable cause, biomedical animal research, and neurobiological research were excluded.

#### Context

This review considered studies conducted in high-income countries (as defined by Organization for Economic Co-operation and Development membership) in any clinical or non-clinical setting. The decision to focus on only high-income countries was based on optimizing comparability in healthcare systems and socioeconomic contexts, as vulvodynia treatment access and healthcare infrastructure vary significantly across countries.^10^

#### Types of sources

Eligible studies included primary research using quantitative, qualitative, or mixed-methods designs, as well as systematic reviews, scoping reviews, and topical reviews. Only studies published after the 2015 ISSVD, International Society for the Study of Women’s Sexual Health, and the International Pelvic Pain Society consensus statement on vulvodynia terminology in 2015^1^ were considered, covering all types of vulvodynia (localized, generalized, provoked, spontaneous, or mixed) with any temporal pattern or onset. Only English-language articles were considered.

### Search strategy

The search strategy aimed to locate published primary studies and reviews that met the inclusion criteria. An initial limited search of MEDLINE (PubMed) and PsycINFO was undertaken to identify articles on the topic. Keywords contained in the titles and abstracts of relevant articles and the index terms used to describe the articles were used to develop a full search strategy. The search strategy, including all identified keywords and index terms, was adapted for each included information source. A definitive search was undertaken in March 2024. Three searches were undertaken, one for each area mentioned in the ‘concept’ (diagnosis and treatment, patient experiences, and healthcare). The terms were searched within each concept and combined with the Boolean connector ‘OR’ and then the searches were combined using the appropriate Boolean connector ‘AND’ to narrow down the search. The following databases were searched: MEDLINE (PubMed), CINAHL, PsycINFO, and Cochrane. The full search strategies are provided in Supplemental Appendix B.

### Study/source of evidence selection

All identified records were collated and uploaded into Rayyan Software (Rayyan Systems Inc.) and duplicates removed.^20^ A partial dual screening of titles and abstracts was conducted, with a second reviewer independently screening 10% of a random sample against the inclusion criteria. Articles appearing to meet the inclusion criteria were reviewed in full by the lead researcher. Any disagreements that arose between the reviewers were resolved through discussion or with a third reviewer. Inter-rater agreement assessed using Cohen’s kappa indicated near perfect agreement among reviewers (*ᴋ* = 0.921, 95% confidence interval = 0.873–0.969). Consistent with the JBI methodology for scoping reviews, a formal quality appraisal of included studies was not conducted, as the primary aim was to map the extent and nature of the existing evidence rather than assess its methodological quality.

### Data extraction and synthesis

Data were extracted from the included articles using custom data extraction tools developed by the authors. A thematic mapping of research areas was undertaken according to the research questions. Each research area was further categorized into different sub-themes and the extracted data were classified and summarized under each sub-theme accordingly. The data extracted included specific details about the participants, type of study, country, methods and key findings relevant to the review questions. Any disagreements that arose between the reviewers were resolved through discussion or through consultation with a third reviewer. Authors of articles were contacted to request missing or additional data, where required.

## Results

### Study inclusion

**Figure 1.**
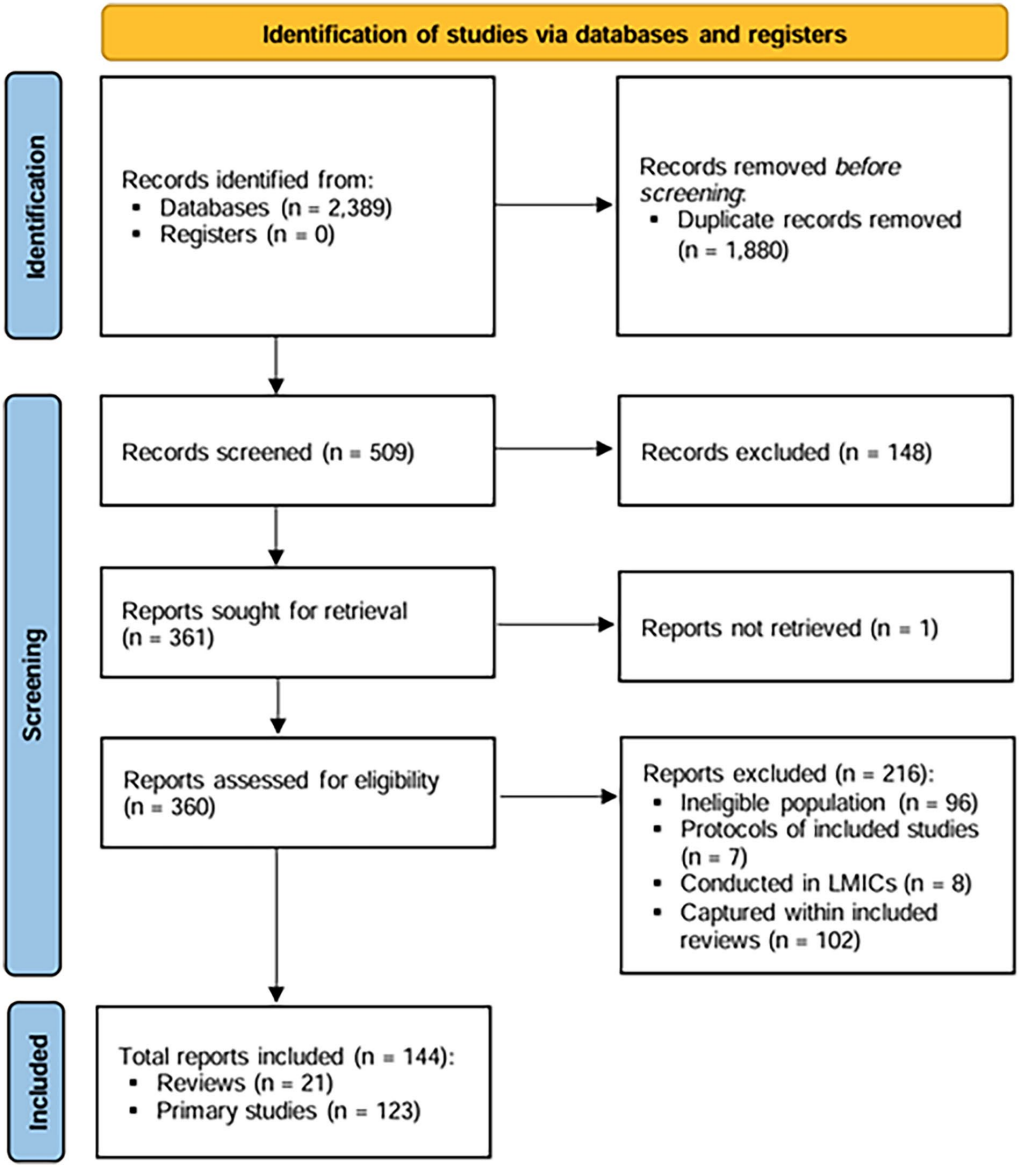
PRISMA flow diagram depicting the study identification and selection process. PRISMA: Preferred Reporting Items for Systematic reviews and Meta-Analyses.

A total of 2389 articles were identified through the search process. After exclusion of 1880 duplicates, titles and abstracts of 509 articles were screened. A total of 148 articles that did not meet the inclusion criteria were excluded at this stage. The full texts of 361 articles were assessed for eligibility, 114 of which were excluded. These articles did not concern vulvodynia, were protocols of included studies, conference abstracts, or were conducted in non-high-income countries. One article was not accessible in full text and so was also excluded. This review did not include a formal quality assessment of the included studies, as the primary objective was to provide an overview of the available literature rather than to evaluate methodological rigor. In total, 246 articles were identified for inclusion, of which 21 were reviews. Of the 225 primary research articles, 102 were captured in the included reviews. Therefore, a total of 21 reviews and 123 primary research articles were included in this review. The study selection process is shown in Figure 1.

### Characteristics of included studies

The majority of the identified reviews were systematic reviews on vulvodynia treatments and interventions. Of the 21 reviews, 12 were systematic reviews: six focused on treatments and outcome measures, and six on psychosocial risk factors, one on the role of nutrition and one on quality of available medical information for patients. Of the remaining nine reviews, five were literature reviews on vulvodynia treatment and four were scoping reviews on treatments and psychosocial barriers for people with vulvodynia. Data extracted from the included reviews (i.e. review type, aim, study characteristics, summary of key findings, limitations, and future recommendations) are presented in Supplemental Appendix C.

Of the 123 primary studies not included in the identified reviews: 29 focused on vulvodynia treatment; 30 related to vulvodynia symptom presentation, characteristics, and risk factors; and 15 were on diagnosis. Additionally, 31 primary studies related to intimate partners, experience of the condition, and psychosocial factors, and 18 studies related to healthcare experiences. Most primary studies were quantitative; specifically, 98 quantitative, 20 qualitative, and 5 mixed-methods studies were identified. Of the 123 primary studies, 86 were conducted in Sweden, the United States, and Canada. Supplemental Appendices D–F provide detailed descriptions of the primary studies.

### Research question 1: What clinical and/or psychosocial research exists on diagnosis and treatment of vulvodynia?

#### Diagnosis

##### Diagnostic and treatment outcome measures

Two systematic reviews^21,22^ examining outcome measurement instruments (OMIs) for provoked vestibulodynia (PVD) highlighted the heterogeneity of outcome measures and their inconsistent application, which reduces the comparability of findings across studies. They also emphasized the lack of instruments assessing psychological impact, functional well-being, and treatment satisfaction. This underscores the need for an internationally agreed-upon, vulvodynia-specific OMI to guide the development of evidence-based treatment guidelines. Several primary studies explored diagnostic measures for vulvodynia. Test–retest reliability of the algometer, vulvalgesiometer, and cotton swab test for vestibular pain, as well as the concurrent validity of the ImageJ software^23^ for determining pain location, were found reliable and valid.^24–26^ The tampon test, though evaluated as a potential measure, was found to underestimate pain severity.^27^ The Vulvar Pain Assessment Questionnaire was developed to diagnose and monitor vulvodynia, demonstrating strong psychometric properties.^28,29^ The Vulvodynia Experience Questionnaire has been also tested, proposing it for clinical trials.^30^ Meanwhile, it was found that the Q-tip test may yield positive results in both women with and without vulvodynia, raising concerns about its diagnostic accuracy.^31^ While many of these studies demonstrated promising psychometric properties, variations in methodology and study quality limit conclusions on validity of findings.

##### Diagnostic subgroups and profiles

Primary research studies on vulvodynia diagnosis have focused on exploring differences between vulvodynia subgroups, such as remission rates between women with primary and secondary vulvodynia, the presence of comorbidities and pain characteristics between diagnostic subgroups, menstrual characteristics of women with vulvodynia, as well as possible racial differences between vulvodynia subtypes.^32–35^ While these studies begin to differentiate clinical profiles, inconsistency in how subgroups are defined and measured makes it difficult to draw firm conclusions or translate findings into clinical practice. The somatosensory profiles of patients with vulvodynia and their pain thresholds as well as pain patterns and pain distribution of the pelvis have been explored showing different distributions of pain for different chronic pelvic pain (CPP) conditions including vulvodynia; however, this could not predict any CPP diagnostic group.^36,37^ This suggests that while somatosensory testing may provide insight into individual pain experiences, it lacks diagnostic specificity. Nocturnal vulval pain with no identifiable cause was explored in prepubertal girls and was argued to be a possible vulvodynia subtype that should be included in the clinical spectrum of night terrors^38^; however, more robust, longitudinal studies are required to validate findings.

##### Symptom presentation and characteristics

Research studies have aimed to better understand the physical and psychological symptoms of women with PVD, assessing pain intensity, duration, and psychological distress. Comparisons between vulvodynia, neuropathic pain, and fibromyalgia have also been made.^39–42^ Clinical profiles of women with chronic pain have been categorized by pain clusters, burden, and distress.^43^ Differences between premenopausal and postmenopausal women with PVD show similar pain symptoms, although postmenopausal women report more burning pain.^41,44^ Studies on pelvic floor and sexual function found no differences in motor function, posture, or breathing patterns,^45^ though sexual dysfunction correlates with age and high pelvic floor muscle (PFM) tone in women, with worse sexual function in women below 30 years old but also above 50 years old.^46^ Symptom characteristics have also been explored in children, adolescents, and between White and Black women with vulval pain.^47,48^ These subgroup analyses suggest that symptom presentation may vary across age, race, and developmental stages, though sample sizes and subgroup definitions vary across studies, limiting comparability. Additionally, research suggests a neuropathic component or altered immune-inflammatory response in vulvodynia^49,50^; however, heterogeneity in study designs and outcome measures precludes clear conclusions about underlying mechanisms. Only one study explored clitorodynia and its impact on sexual function,^51^ suggesting a need for further research into underrepresented subtypes.

##### Risk factors and comorbidities

A systematic review examined the relationship between weight, nutritional status, and metabolic disorders in vulvodynia but found no conclusive results due to a lack of data and low-quality studies.^52^ Critical factors like BMI, nutritional status, and weight fluctuations were often missing and overlooked in clinical settings, reflecting gaps in assessment and reporting consistency that limit confidence in findings.

Another review explored the connection between vulvodynia and vulvovaginal candidiasis, finding that women with PVD were more likely to report a history of candidiasis.^53^ However, the causal relationship remains unclear due to reliance on self-reported symptoms and low-quality studies. They also highlighted an altered cytokine response in women with PVD compared to healthy controls, though the cause remains uncertain. Factors like pelvic floor hyperactivity and low lubrication may be responsible for this. They emphasized the need for more robust studies while also arguing that a PVD diagnosis should be considered in women with self-reported vulvovaginal candidiasis.^53^ While these findings point to possible immunological and infectious contributors, the lack of consistent diagnostic confirmation and reliance on retrospective reporting weaken the strength of associations.

Regarding other risk factors for vulvodynia, primary research studies have found positive associations between sexual and physical abuse, pain history, adverse urinary symptoms, past environmental exposures, as well as hygienic behaviors and vulvar sensitivity.^54–63^ The epidemiological characteristics, comorbidities, and risk factors associated with vulvodynia in Spain have shown similarities with other countries.^64^ Possible comorbidities have also been explored, with dysmenorrhea and dyspareunia (painful intercourse) identified as conditions that may co-occur with vulvodynia.^65,66^ Birth-related factors and genetic predisposition have been explored and shown to be associated with the development of vulvodynia.^67,68^ Collectively, these studies highlight a range of biopsychosocial factors potentially contributing to vulvodynia. However, methodological limitations preclude firm conclusions.

#### Treatment

##### Comparative reviews of treatments

Reviews on available treatment options and interventions have examined improvement in pain reduction, dyspareunia symptoms, as well as sexual function. A systematic review showed that multimodal physiotherapy, when compared with lidocaine treatment, is effective for improving pain and dyspareunia symptoms.^69^ Similarly, multimodal physiotherapy has shown some evidence of effectiveness for reducing pain and dyspareunia.^70^ Nevertheless, a systematic review and meta-analysis showed that oral desipramine plus topical lidocaine or oral desipramine alone was the only intervention that significantly improved sexual function; however, they did not find improvements on dyspareunia or daily vestibular pain from any other interventions.^71^ Notably, the reviews highlighted the lack of rigorous, extensive research and methodological shortcomings, which impair generalizability and validity. They stressed the need for standardized measures to assess sexual function and satisfaction, along with more detailed evaluations of clinical complaints.

##### Pharmacological treatments

Reviews on pharmacological treatments for vulvodynia have examined oral and topical therapies. A scoping review found enoxaparin to be the only treatment that consistently reduced pain, while botulinum toxin (BoNT) showed mixed results.^72^ Gabapentin was the best-tolerated oral medication, and topical amitriptyline caused the least skin irritation.^72^ In another review, tricyclic antidepressants and gabapentin were identified as the most common treatments.^73^ Both reviews noted that the low quality of studies limits conclusions on effectiveness, despite generally positive outcomes. Issues such as variations in pain assessment, small sample sizes, and inconsistent dosing contribute to this uncertainty, along with a lack of evidence on combining pharmacological treatments with other modalities. BoNT was also reviewed for its effect on CPP, including vulvodynia, and found lower doses safe and effective. However, the study grouped vulvodynia with other pain conditions, emphasizing the need for rigorous randomized controlled trials (RCTs) to refine dosing and techniques.^74^

Primary studies have shown positive results for treatments like amitriptyline 0.5% and oestriol 0.03% in organogel (AOO), enoxaparin, and low-dose sinecatechins ointment.^75–77^ Racial differences in gabapentin efficacy were observed, with White women responding better than Black women.^78^ Studies on abobotulinumtoxinA showed safety and positive effects on sexual function and psychosocial outcomes,^79^ and transvaginal BoNT injections were effective in adolescent case studies.^80^

##### Physiotherapy

Two reviews examined the efficacy of physical therapy for improving dyspareunia, quality of life, and sexual function in women with PVD and generalized vulvodynia.^81,82^ They reviewed interventions like biofeedback, dilators, electrical stimulation, shockwave, pelvic floor exercises, education, and multimodal physical therapy. Multimodal approaches, including education on pain management, PFM training, and home exercises, were found most effective for improving pain and sexual function. Biofeedback showed long-term benefits for sexual function among isolated therapies. However, the reviews highlighted high variability in dosages, treatment duration, and a lack of rigorous RCTs, making it difficult to draw conclusions.^81,82^

Primary research studies have also assessed physical therapy for PVD, focusing on education, pelvic floor exercises with biofeedback, and manual therapy. These approaches, along with spinal cord stimulation and holistic biofeedback, demonstrated positive effects on pain reduction and sexual function.^83,84^ Transcutaneous electrical nerve stimulation and low-intensity shockwave therapy also showed promise in reducing pain and dyspareunia.^85^ Research on somatocognitive therapy indicated it as an acceptable multimodal treatment, with participants reporting positive experiences.^86–88^

##### Laser treatment

A scoping review of laser treatments for vulvodynia, including surgical ablative, micro-ablative, non-ablative, and photobiomodulation types, was identified.^89^ They found a lack of RCTs, with most evidence deriving from case reports, hence hindering conclusions on the effectiveness of laser treatment for vulvodynia. They also highlighted the problematic use of the umbrella term regarding lasers as it encompasses a large variety of different devices and safety levels. Although technological advances have improved safety with less invasive lasers being used, their effectiveness in improving pain symptoms remains unclear. Notably, some studies have questioned the clinical utility of CO_2_ laser treatment, further underscoring the need for higher-quality evidence, clearer diagnostic subcategories, and additional research on non-invasive options like photobiomodulation.^89^

Primary research studies on laser therapy have examined and supported the efficacy and safety of the CO_2_ fractional laser using self-reported measures and the cotton swab test on pain improvement, as well as the feasibility and acceptability of high-intensity laser therapy on pain improvement during intercourse and patient satisfaction for women with PVD.^90^

##### Acupuncture

The identified primary studies on acupuncture focused on evaluating acupuncture treatment protocols on pain-related outcomes and sexual function using questionnaires and the cotton swab test that could be used in clinical trials.^91,92^ One RCT study explored how to successfully implement blinding and randomization, with another study exploring what motivated women to take part in acupuncture trials, highlighting reducing pain and healthcare costs as the main motivating factors.^93,94^

##### Psychological therapies

One review aimed to provide a detailed overview of cognitive behavioral therapy (CBT) and mindfulness-based approaches in treating PVD.^95^ Most included studies focused on CBT, which was suggested to improve sexual function, pain symptoms, and psychosexual outcomes. Nevertheless, the quality of the included studies is not clear. Only one study on mindfulness as a potential treatment for vulvodynia was identified, and authors underlined the need for more research on mindfulness-based cognitive therapy (MBCT) with rigorous trials and head-to-head studies comparing the efficacy of CBT versus MBCT.^95^ They emphasized the need to investigate specific cognitive and affective variables as predictors of pain outcomes and the mechanisms of therapeutic change that underlie the MBCT approach.^95^

Recent primary RCT studies have explored the feasibility and effectiveness of online acceptance and commitment therapy (ACT) for women with PVD. ACT has been assessed in terms of feasibility, pain-related outcomes and behaviors, sexual functioning, as well as cost-effectiveness, showing positive outcomes for women with PVD in addition to receiving usual care^96–100^; however, most studies are limited by small sample sizes and short follow-up periods, making it difficult to assess long-term efficacy. Another study explored the role of CBT with partner involvement as treatment for women with PVD in a relationship on self-reported measures of pain, psychosocial outcomes, sexual function and treatment satisfaction showing positive outcomes on improved sexual functioning, pain catastrophizing, and avoidance for some participants.^101^ While initial findings are encouraging, further research is needed to clarify for whom and under what circumstances psychological interventions are most effective.

##### Multidisciplinary interventions and other treatments

Two reviews were identified on multidisciplinary therapy and the effectiveness of multidisciplinary interventions for vulvodynia. One review aimed to explore clinical trials on vulvodynia to develop strategies for individualizing multidisciplinary therapy for vulvodynia.^102^ They emphasized four key characteristics to structure multidisciplinary therapy models – specifically, the level of pain, sexual functioning, PFM tone, and vulvodynia subtypes (primary or secondary) – arguing that therapeutic modalities should be applied based on the different subgroups of women.^102^ Similarly, a scoping review argued that structured interdisciplinary management programs are promising treatments, also highlighting superior outcomes for non-pharmacological treatments for localized PVD compared to pharmacological treatments.^103^ Nevertheless, both reviews highlighted the methodological shortcomings of the included studies, such as the lack of robust samples and RCTs designs. Additionally, it was emphasized that the high heterogeneity of pain measurement tools, ranging from cotton swab tests, tampon test, insertion of a finger or vaginal dilators, attempted vaginal penetration, and vulvalgesiometers, as well as a variety of pain questionnaires, limits generalizability.^102^

The identified primary research studies on other treatments concern the positive effect of a multidisciplinary treatment on pain reduction and psychosocial outcomes in a case study, an osteopathic manipulative treatment effective on reducing pain and depression, the efficacy of peripherally and centrally acting medications currently used in clinical practice, as well as the preoperative and postoperative changes and improvements of patients following a modified vestibulectomy.^104–107^

### Research question 2: What clinical and/or psychosocial research exists on patient experience of vulvodynia?

#### Experience with vulvodynia

One systematic review was identified aiming to explore qualitative studies on the experiences of women living with vulvodynia.^14^ They argued that vulvodynia has immense negative consequences on women’s lives, particularly on their sexual identity, femininity, and self-esteem. Consequently, vulvodynia adversely affects personal and social relationships leading to experiences of shame and guilt and self-blame. Importantly, they highlighted the importance of multidisciplinary approaches to managing vulvodynia to focus on helping women feel in control of their pain experience.^14^ However, the synthesis included a limited number of studies, and most participants were young, White, heterosexual women, limiting the generalizability of findings. Authors argued that more research should be conducted on individuals with different gender identities and women from different ethnic and cultural backgrounds, asexual, bisexual, and lesbian women, and women who are postmenopausal. They encouraged sexual health and sexuality to be discussed in clinical settings to combat stigma and taboo ideologies.^14^

Primary qualitative studies using interviews with heterosexual women have explored the experience of living with vulvodynia, revealing its predominantly negative impact on gender identity and intimate relationships, but also highlighting some positive effects on self-advocacy.^108–110^ In another study, pain experiences and pain management strategies of women were also explored, identifying possible high-risk behaviors, such as high alcohol use, to manage pain symptoms.^111^ While these qualitative accounts provide valuable insights into lived experience, broader sampling and consistent use of intersectional frameworks would enhance our understanding of how sociocultural factors shape the experience of vulvodynia.

#### Psychosocial factors

Three reviews explored psychosocial factors in vulvodynia. One review emphasized the importance of intimacy and intercourse-related processes alongside emotional outcomes like anxiety, depression, and somatization to understand vulvodynia experiences.^112^ Another review identified psychosocial barriers people with vulvodynia may face, such as fear, anxiety, catastrophizing, adverse childhood events, and low self-efficacy, along with structural barriers like diagnostic delays, stereotyping, lack of knowledge, and high healthcare costs.^10^ A systematic review and meta-analysis found a strong association between adverse psychological outcomes and vulvodynia, suggesting it should be treated through a biopsychosocial lens.^113^

However, the reviews noted that most research focuses on PVD, with limited studies on generalized vulvodynia and its subtypes. They highlighted gaps in theoretical frameworks for treatments and insufficient research on the causal relationship between psychosocial factors and vulvodynia. Inclusivity was lacking, particularly for underrepresented populations and those with physical comorbidities such as Ehlers–Danlos syndrome. The reviews called for more robust studies, particularly on follow-up psychological evaluations post-treatment.^10,111,112^ Taken together, these reviews point to consistent psychosocial correlates of vulvodynia, but the predominance of cross-sectional designs and limited diversity in sampling constrain causal inferences and generalizability.

Primary studies examined the impact of distress, fatigue, illness perceptions, and cognitive-behavioral factors on pain severity and interference. Psychological flexibility, body exposure anxiety, and avoidance during sexual intercourse have been linked to pain and sexual dysfunction.^13,114,115^ Another study explored the moderating role of solicitous partner responses on penetrative pain.^116^ Other studies reported higher rates of perfectionism, self-compassion deficits, and impostor syndrome in women with PVD.^117,118^ While these findings offer insight into relevant psychological mechanisms, most studies rely on self-report measures and small samples, with limited longitudinal data to establish directionality.

#### Intimate relationships and partner role

Most partner research has focused on women with PVD in heterosexual relationships, examining couples’ experiences with vulvodynia. Studies have explored male partner responses to pain, sexual intimacy, relationship satisfaction, and communication patterns using self-report questionnaires and interviews.^119–123^ This focus has generated valuable insights into dyadic coping and relational dynamics, though it provides limited understanding of diverse relationship contexts, including same-sex couples and gender-diverse individuals.

Research on relationship and sexual satisfaction has addressed male partners’ perceptions of women’s pain and communication styles. Collaborative sexual communication and empathetic responses to women’s pain have been highlighted as crucial for improving relationship satisfaction.^124–126^ Partner supportiveness, particularly in individuals of color, was associated with reduced distress and dissatisfaction regarding sexual function.^127^ However, much of this work is cross-sectional, limiting our understanding of how partner dynamics may influence symptom trajectories over time.

Studies have also investigated partner pain-related cognitive variables, like pain catastrophizing, and their impact on women’s pain intensity and emotional outcomes such as depression and anxiety.^128,129^ Women’s sexual assertiveness has been found to mediate the positive effects of facilitative partner responses on sexual function.^130^ Additionally, couples’ cognitive variables, such as pain catastrophizing, have been identified as potential mediators in cognitive-behavioral couple therapy.^131^ Relational and psychological processes within couples appear closely linked to pain outcomes, highlighting the potential utility of dyadic interventions.

#### Pregnancy

Retrospective research on vulvodynia and pregnancy has aimed to compare reproduction and mode of delivery between women with a vulvodynia diagnosis and controls, as well as to evaluate obstetric outcomes of women with vulvodynia.^132,133^ Another study explored changes of pain symptoms from pregnancy to postpartum and the associated emotional outcomes, including fear of childbirth, arguing that mode of birth may influence symptom trajectory.^133^ Importantly, a primary study investigated how women’s chronic pain affected clinicians’ examination and management during labor as well as postpartum care. This study identified differences between care providers in antenatal, labor, and postnatal care settings, and significant knowledge gaps and variability in practice,^134^ underscoring the need for improved provider education and clearer clinical guidelines for managing vulvodynia in perinatal care.

### Research question 3: What clinical and/or psychosocial research exists on seeking/accessing/receiving care for vulvodynia?

A systematic review evaluated the quality of information on the internet for patients with vulvodynia. Most articles derived from the United Kingdom, United States, and Australia, and were shown to have low credibility, reliability, and accuracy scores, as well as low readability.^135^ They argued that more rigorous and accurate information should be available to vulvodynia patients to control for discrepancies between information from healthcare professionals and information obtained online, which can damage trust between patient and clinician, impact willingness to accept recommended treatment, and can cause significant harm.^135^ Similarly, in Spain, the quality, content, and readability of websites providing health information on vulval pain, including vulvodynia, have been evaluated. Findings indicated that these resources were generally of low quality and difficult for patients to interpret,^136^ underscoring the need for higher-quality, evidence-based patient education resources.

Primary research studies on seeking and accessing healthcare for vulvodynia have focused on both women and healthcare professionals. In particular, the experiences of women diagnosed with vulvodynia as well as the experiences of women seeking care for vulvovaginal symptoms prior to receiving a diagnosis have been explored, revealing a pattern of negative experiences for women both before and after receiving a diagnosis of vulvodynia.^16,137–141^ Additionally, women’s experiences with clinician-led management of vulvodynia, including recommended treatments, care received, and the role of partner support in help-seeking, have been investigated. Findings highlight significant variability in treatment approaches, resources, and clinical practices, as well as a lack of continuity in care.^121,142–144^ Most studies have used online surveys and interviews to explore the healthcare-seeking experiences of women with vulvodynia, mainly in the United Kingdom, Australia, and North America. Of note, there was a heavy reliance on self-selected samples, which may have introduced bias into these findings.

Moreover, research studies have also focused on vulvodynia awareness and knowledge among student healthcare providers, the professional experiences and practices of therapists and the challenges they face, as well as on the barriers of general practitioners in the diagnostic process of women with vulvovaginal symptoms, such as stigma, negative emotional experiences, and poor knowledge of vulvodynia.^145–149^ The impact of rurality on clinicians’ knowledge, diagnosis, and management of vulvodynia has also been explored in Canada and has been shown to also negatively impact vulvodynia diagnosis and management.^150^ Finally, another study explored difficult conversation topics (e.g. sexual intercourse) between vulvodynia patients and their romantic partners, offering guidance to clinicians in counseling.^151^

## Discussion

### Summary of key findings

This review aimed to provide a comprehensive overview of the nature and extent of existing clinical and psychosocial research on vulvodynia. A substantial number of reviews and primary studies were identified, with most focusing on treatment and diagnosis, and fewer addressing patient experiences and healthcare access. Nevertheless, the findings of the included reviews highlight that most studies are of low quality, characterized by a lack of rigorous study design and heterogeneity of outcome measures, treatment applications, and dosages. The variability of outcome measures is also evident when examining the methodology of primary studies (see Supplemental Appendix C). This hinders comparability across studies, replication of findings, and, most importantly, the ability to draw robust conclusions about treatment effectiveness. Most research is focused on women with PVD, the most common subtype, with some addressing generalized vulvodynia and only one study examining clitorodynia; however, most studies included in this review do not differentiate between subtypes of vulvodynia or clarify whether participants were officially diagnosed or self-diagnosed. Future research should address these methodological shortcomings by utilizing standardized diagnostic criteria and outcome measures to improve the consistency and quality of evidence on vulvodynia and its treatment effectiveness and satisfaction.^1,152^

### Interpretation of findings

Although vulvodynia remains a largely underdiagnosed and under-recognized condition, a substantial body of research has explored its treatment and management. Positive outcomes have been reported for gabapentin, oral desipramine combined with topical lidocaine, and enoxaparin in reducing pain and dyspareunia.^72,73^ Similarly, lower doses of BoNT, multimodal physiotherapy incorporating pain management education, PFM training, and home exercises, as well as biofeedback, have shown potential benefits for patients.^74,81,82^ Individualized multidisciplinary interventions have been proposed as the gold standard for managing vulvodynia, given the complexity and variation in its etiology.^115^ However, their design and implementation in clinical practice remain unclear. Multidisciplinary approaches often include psychological treatments, as vulvodynia is associated with adverse psychological outcomes such as anxiety and depression. Despite this, research on mindfulness, ACT, and CBT for vulvodynia remains scarce.^98,99^ More broadly, the limited strength of evidence across many treatment modalities appears to be driven by several persistent methodological challenges: small sample sizes, lack of RCTs, high variability in treatment protocols, and inconsistent outcome measures. These limitations restrict the generalizability of findings and make it difficult to draw robust conclusions about the efficacy of specific interventions.

Although a wide variety of treatment approaches exist, standardized clinical guidelines for vulvodynia remain absent. This can be largely attributed to the lack of high-quality evidence, stemming from methodological limitations and heterogeneity in treatment protocols and dosages, as noted in most included reviews. Furthermore, inconsistent outcome measures across studies impede comparisons and hinder definitive conclusions about treatment efficacy, a persistent issue in vulvodynia research. The multifactorial etiology of vulvodynia and the heterogeneity of its presentation pose further significant challenges to developing and implementing standardized treatment guidelines. This heterogeneity extends to patients’ treatment goals, which may vary widely depending on the nature and impact of their symptoms. Nonetheless, standardized guidelines remain important for supporting consistent and equitable care, particularly in primary care settings where clinicians may have limited expertise in vulvovaginal pain conditions.^153^ An individualized approach to treatment is essential, emphasizing the need to identify the underlying causes of vulvodynia to develop appropriate treatment options and evaluate their effectiveness. Well-developed guidelines can facilitate patient-centered care by offering a structured framework that still allows for flexibility in tailoring treatment plans to individual needs and goals.

Clinical disagreement around the classification and clinical relevance of vulvodynia subtypes may further impede consensus on treatment guidelines. Although subcategories have been proposed and are already used in some clinical contexts, there is not yet universal agreement on their diagnostic validity or utility. This lack of standardization in classification systems likely contributes to variability in treatment approaches and creates additional barriers to guideline development. The importance of establishing diagnostic subcategories for vulvodynia has been previously highlighted.^70^ Although not yet validated, subcategories currently used in clinical settings to guide management include hormonally associated vestibulodynia, inflammatory vestibulodynia, congenital and acquired neuroproliferative vestibulodynia, and overactive (hypertonic) PFM dysfunction.^70^ Validating these subcategories and integrating them into clinical practice may further enhance the precision and consistency of vulvodynia management.

Research on diagnostic measures for vulvodynia is similarly varied. A variety of diagnostic measures have been used, but their effectiveness and accuracy remain uncertain. For example, while Q-tip tests are commonly used to diagnose vulvodynia, positive Q-tip tests have been reported in women without vulvodynia symptoms.^31^ Additionally, the tampon test may underestimate pain symptoms.^27^ Research on diagnostic subcategories based on etiology could inform the development of more accurate diagnostic measures, and vice versa, supporting a more reliable diagnostic framework for vulvodynia. Standardized diagnostic measures would improve the consistency of clinical diagnoses, enable better comparisons across studies, and ultimately enhance treatment approaches for affected individuals.

Several studies have examined risk factors associated with the development of vulvodynia and its outcomes. Associations with sexual and physical abuse, pain history, adverse urinary symptoms, environmental exposures, hygienic behaviors, vulvar sensitivity, and potential genetic predisposition have been identified. However, key gaps remain, particularly regarding the roles of nutrition, diet, vulvovaginal candidiasis, and comorbidities such as fibromyalgia, Ehlers–Danlos syndrome, dermatological conditions, and attention deficit hyperactivity disorder.^52,53,154^ While psychosocial factors such as fear, anxiety, and depression have been investigated, differences and similarities across vulvodynia subgroups and the role of theoretically based treatments grounded in psychosomatic medicine remain unexplored.^13,115^ Research on the role of intimate partners highlights their critical influence, as partners’ responses to women’s pain and communication within the couple are associated with distress, sexual function, and satisfaction. However, it remains unclear how partners can best support women in managing vulvodynia.

Cumulatively this literature suggests the lack of standardized treatment guidelines and diagnostic measures, coupled with the under-recognition of vulvodynia and the stigma associated with the condition, significantly impacts healthcare access and patient experiences. Healthcare professionals often demonstrate confusion, limited knowledge, insufficient education, and a lack of understanding, empathy, and continuity of care. These challenges are not only a result of individual clinician factors but also reflect broader systemic issues. Medical education rarely includes training on vulvovaginal pain conditions, leading to a persistent knowledge gap among frontline healthcare providers.^147^ In addition, funding priorities often neglect chronic, gendered pain conditions such as vulvodynia,^18,155^ limiting the development of educational resources, clinical services, and research. Systemic biases, including the normalization of women’s pain, discomfort with discussing sexual health, and disparities in care experienced by racial and sexual minorities, may further contribute to delayed diagnosis, mismanagement, and inadequate support.^10,121,137,156^ In addition, the quality of patient-facing resources on vulvodynia is often poor, with many being difficult to understand or lacking evidence-based guidance, further compounding the challenges patients face when seeking information and support.^135,136^ Addressing these challenges will require a multifaceted approach, including improving the quality and accessibility of patient information, expanding available treatment and pain management options, increasing education and awareness among healthcare professionals, and establishing a clear, coordinated pathway of care. Future work should also consider how to address these structural barriers through curriculum reform, provider training, and policy-level interventions that elevate the visibility and legitimacy of vulvodynia within healthcare systems.

### Strengths and limitations

This review provides a comprehensive synthesis of available clinical and psychosocial evidence on vulvodynia, including review articles and primary studies, and highlights several key issues. We followed JBI methodological guidance for scoping reviews and adhered to PRISMA-ScR reporting guidelines to ensure methodological rigor and transparent reporting.^19,157,158^ However, some limitations must be noted. Biomedical and translational studies were excluded. While such research is critical for advancing understanding of vulvodynia’s etiology and informing treatment development, its exclusion reflects the methodological focus of this review on applied clinical and psychosocial research. The review was also restricted to studies published since 2015, reflecting the adoption of updated vulvodynia terminology. While earlier research has been pivotal in advancing the field, this focus ensured alignment with the most current definitions and frameworks. Furthermore, only studies published in English from high-income countries were considered. This was intended to optimize comparability across studies, given significant differences in healthcare infrastructure, diagnostic practices, and treatment availability between high-income and low- and middle-income countries (LMICs). However, this may have excluded valuable insights from LMICs, where cultural, economic, and structural differences could influence the experience, diagnosis, and treatment of vulvodynia. Future reviews should consider including research from LMICs to capture a more globally representative understanding of vulvodynia and to explore how healthcare access, stigma, and treatment approaches may differ across diverse healthcare systems.

Given the large volume of relevant studies identified, we synthesized findings from relevant review articles rather than extracting data from each of the primary studies they included. This approach enabled us to map the existing literature efficiently and transparently, while ensuring the review remained feasible within the constraints of our available time, personnel, and funding. While we acknowledge that reviews may not always fully capture the nuances of individual studies, their inclusion was appropriate for our scoping methodology, which prioritizes breadth of coverage and mapping of the field over detailed appraisal of every individual study. Additionally, given the volume and variety of studies included, the quality of primary studies was not assessed, limiting detailed discussion of their findings. However, some included reviews provided quality assessments, offering insight into the methodological limitations of this literature. Future reviews should comprehensively assess the quality of evidence to better evaluate the veracity of findings.

### Future research directions

#### Methodological rigor and outcome standardization

Future research on vulvodynia should address existing methodological limitations and prioritize robust, theory-driven study designs. This includes the need for rigorous clinical trials and consistent use of validated outcome measures. Achieving international consensus on vulvodynia-specific outcome measures is crucial, alongside the development of standardized tools to assess psychological impact, functional well-being, and treatment satisfaction, and ensuring their consistent implementation across research and clinical practice. The recent publication of a core outcome set for treatment studies for PVD^159^ represents a significant advancement toward this goal.

#### Diagnostic subgrouping and mechanisms

A key focus for future research should be the development and validation of diagnostic subcategories based on etiology, which may enhance understanding, guide clinical decision-making, and support the development of standardized treatment guidelines.^70^ Further investigation into hormonal influences, particularly menstrual and menopausal characteristics, as well as comorbidities and risk factors (e.g. pudendal neuralgia, lichen sclerosus, spinal pathology, vulvar dysesthesia, and persistent genital arousal disorder) is also warranted. Psychological distress and psychosocial factors may serve as meaningful diagnostic classifiers and should be more fully explored as contributors to vulvodynia heterogeneity.

#### Psychological treatments and mechanisms

There is a pressing need for research into psychological treatments, including psychotherapy and digital interventions, with particular attention to cognitive and affective predictors of pain outcomes. Follow-up evaluations and long-term effectiveness data are notably lacking and should be prioritized in future trials.

#### Diversity, inclusion, and patient involvement

Future studies should broaden their scope to include individuals from diverse gender and sexual identities, relationship structures beyond heterosexual partnerships, and underrepresented populations. Greater involvement of patients in shaping research (e.g. identifying priorities and core outcomes) is critical to ensuring that future studies reflect the lived realities, values, and needs of those most affected by vulvodynia.

## Conclusion

Research on vulvodynia has advanced considerably over the past decade; however, the condition remains largely under-recognized, with individuals affected by vulvodynia often facing adverse outcomes and negative experiences when seeking care. Despite notable research efforts, significant methodological shortcomings persist in the literature. Future research should prioritize rigorous clinical trials, the identification of both clinical and patient-reported outcomes as indicators of treatment response, and the standardization of outcome measures. Furthermore, developing and tailoring treatment plans to the diverse subtypes of vulvodynia is essential to improving management and care.

## Supplemental Material

sj-docx-1-whe-10.1177_17455057251345946 – Supplemental material for A scoping review of vulvodynia research: Diagnosis, treatment, and care experiences Supplemental material, sj-docx-1-whe-10.1177_17455057251345946 for A scoping review of vulvodynia research: Diagnosis, treatment, and care experiences by Athina Zoi Lountzi, Purva Abhyankar and Hannah Durand in Women's Health

sj-docx-2-whe-10.1177_17455057251345946 – Supplemental material for A scoping review of vulvodynia research: Diagnosis, treatment, and care experiences Supplemental material, sj-docx-2-whe-10.1177_17455057251345946 for A scoping review of vulvodynia research: Diagnosis, treatment, and care experiences by Athina Zoi Lountzi, Purva Abhyankar and Hannah Durand in Women's Health

sj-docx-3-whe-10.1177_17455057251345946 – Supplemental material for A scoping review of vulvodynia research: Diagnosis, treatment, and care experiences Supplemental material, sj-docx-3-whe-10.1177_17455057251345946 for A scoping review of vulvodynia research: Diagnosis, treatment, and care experiences by Athina Zoi Lountzi, Purva Abhyankar and Hannah Durand in Women's Health

sj-docx-4-whe-10.1177_17455057251345946 – Supplemental material for A scoping review of vulvodynia research: Diagnosis, treatment, and care experiences Supplemental material, sj-docx-4-whe-10.1177_17455057251345946 for A scoping review of vulvodynia research: Diagnosis, treatment, and care experiences by Athina Zoi Lountzi, Purva Abhyankar and Hannah Durand in Women's Health

sj-docx-5-whe-10.1177_17455057251345946 – Supplemental material for A scoping review of vulvodynia research: Diagnosis, treatment, and care experiences Supplemental material, sj-docx-5-whe-10.1177_17455057251345946 for A scoping review of vulvodynia research: Diagnosis, treatment, and care experiences by Athina Zoi Lountzi, Purva Abhyankar and Hannah Durand in Women's Health

sj-docx-6-whe-10.1177_17455057251345946 – Supplemental material for A scoping review of vulvodynia research: Diagnosis, treatment, and care experiences Supplemental material, sj-docx-6-whe-10.1177_17455057251345946 for A scoping review of vulvodynia research: Diagnosis, treatment, and care experiences by Athina Zoi Lountzi, Purva Abhyankar and Hannah Durand in Women's Health
