# Supplementary material for: A scoping review of vulvodynia research: Diagnosis, treatment, and care experiences
Source: Womens Health (Lond). 2025 Jun 17;21:17455057251345946. doi: 10.1177/17455057251345946 (PMC12174717; doi:10.1177/17455057251345946)
Supplement: sj-docx-2-whe-10.1177_17455057251345946 – Supplemental material for A scoping review of vulvodynia research: Diagnosis, treatment, and care experiences [file sj-docx-2-whe-10.1177_17455057251345946.docx]

**Appendix A - Search Strategy**

| Condition | Operator | Concept |
| --- | --- | --- |
| Vulvodynia [MeSH] OR  Vestibulodynia [MeSH] OR  Clitorodynia [MeSH] | **AND** | **[1] Medical/Clinical research**  Diagnosis [MeSH] OR  Treatment [MeSH] OR  Therapy [MeSH]  Evaluation OR  Management OR  Multidisciplinary examination OR  Epidemiology OR  Prevalence OR  Recurrence  Aetiology/etiology OR  Prognosis OR  Risk factors OR  Interventions OR  Physiopathology OR  Pharmacological/ non-pharmacological approaches    **[2] Psychosocial research / Patient experience**  Anxiety [MeSH] OR  Depression [MeSH] OR  Psychology OR  Psychosocial OR  Stigma/ Stereotype OR  Intimacy OR  Well-being OR  Sexual satisfaction OR  Sexual motivation OR  Sexual function OR  Partner OR  Partner response OR  Relationship satisfaction OR  Couple OR  Experience OR  Somatisation OR  Stress OR  Mindfulness OR  Counselling OR  Acceptance and Commitment Therapy OR  Cognitive Behavioural Therapy    **[3] Psychosocial research/ Healthcare**  Patient Acceptance of Health care [MeSH] OR  Acceptability of Healthcare [MeSH] OR  Availability of Healthcare [MeSH] OR  Healthcare Costs [MeSH] OR  Health Services Accessibility [MeSH] OR  Education OR  Awareness OR  Knowledge OR  Patient care OR  Health seeking OR  Healthcare experience OR  Primary healthcare |
|  |  |  |
