# Supplementary material for: A scoping review of vulvodynia research: Diagnosis, treatment, and care experiences
Source: Womens Health (Lond). 2025 Jun 17;21:17455057251345946. doi: 10.1177/17455057251345946 (PMC12174717; doi:10.1177/17455057251345946)
Supplement: sj-docx-3-whe-10.1177_17455057251345946 – Supplemental material for A scoping review of vulvodynia research: Diagnosis, treatment, and care experiences [file sj-docx-3-whe-10.1177_17455057251345946.docx]

| **Appendix B – Data Extraction Table for Reviews** | | | | | | |
| --- | --- | --- | --- | --- | --- | --- |
| **Study ID** | **Review Type** | **Aim** | **Study characteristics** | **Review Findings** | **Review Limitations** | **Author Recommendations** |
| Dunkley & Brotto, 2016 | Topical Review | To provide a detailed overview of CBT and mindfulness-based approaches for treating PVD | Research studies on CBT and MBCT as treatments for PVD | - Strong evidence supports the efficacy of CBT for PVD - Limited evidence available for brief mindfulness-based interventions | - Non-systematic search strategy - Only one relevant study on mindfulness-based interventions was identified | - Further research comparing CBT and MBCT efficacy - Identify predictors of pain outcomes for CBT/MBCT in PVD |
| Morin et al., 2017 | Systematic Review | To assess the effectiveness of physical therapy in reducing pain and improving sexual function in PVD | RCTs, cohort studies, and case reports on physical therapy modalities for women with PVD | Biofeedback, dilators, electrical stimulation, education, multimodal therapy, and multidisciplinary approaches effectively reduced pain and improved sexual function | Few RCTs and reliance on high-bias non-RCT studies. | Conduct robust RCTs with validated outcome measures, adequate samples, and long-term follow-ups to confirm effectiveness |
| Nascimento et al., 2024 | Systematic Review | To assess the efficacy of physiotherapy for vulvodynia | Studies on EMG biofeedback, TENS, shockwave therapy, physiotherapy, and pelvic floor exercises for vulvodynia (vestibulodynia / generalised) | - All interventions reduced pain and improved quality of life and sexual function. - Multimodal physiotherapy showed the greatest potential. | Small number of quality studies and heterogeneity among them. | Develop core outcome sets for vulvodynia to enable systematic analyses and well-designed trials |
| Bohm-Starke et al., 2022 | Systematic Review | To review RCTs and non-RCTs of interventions for PVD | Trials of pharmacological, surgical, psychosocial, and / or physiotherapy interventions, either individual or group-based, for premenopausal women with PVD | Multimodal physiotherapy compared with lidocaine treatment was the only intervention with some evidential support | Low certainty of evidence precludes conclusions on intervention effects | Conduct stringent trials and define core outcome sets for PVD |
| Sadownik et al., 2018 | Systematic Review | To identify outcome measures used in vulvodynia treatment studies for adult women and categorise them using the six IMMPACT core outcome domains | RCTs and non-RCTs focused on women with PVD and / or generalised vulvodynia | Significant variation in metrics, instruments, and clinician-reported outcomes makes comparing vulvodynia clinical trials challenging | Exclusion of non-English articles and conference abstracts | Improve reporting of outcomes to facilitate data comparison and pooling |
| Davenport et al., 2018 | Systematic Review | To detail the outcome measurement instruments for therapeutic interventions for PVD | RCTs and observational studies investigating therapeutic interventions for PVD | Large heterogeneity in therapeutic outcomes and inconsistency in their application | Exclusion of non-English articles and conference abstracts | - Develop a standardised set of core outcome measures and validated instruments - Develop a multidimensional vulvodynia-specific outcome tool that incorporates IMMPACT recommendations and sexual functioning |
| Pérez-López et al., 2019 | Systematic Review and Meta-Analysis | To quantify the effects of available treatments of vestibulodynia | RCTs comparing therapeutic interventions to control, sham, or placebo in women with vulvodynia, vestibulodynia, or vulvar vestibulitis | Oral desipramine (with or without lidocaine) improved sexual function, but most interventions showed no improvement in vestibulodynia | Scarcity of studies, small patient numbers, and heterogeneity of treatments and outcomes | Conduct larger studies with standardized outcomes and detailed assessments |
| Loflin et al., 2019 | Topical Review | To evaluate the literature on pharmacological treatment options for vulvodynia | Research studies on oral and / or topical therapies for vulvodynia / vestibulodynia / vulvar vestibulitis | - Oral tricyclic antidepressants and gabapentin are the most used treatments for vulvodynia pain - Topical treatments with efficacy data include amitriptyline, gabapentin, lidocaine, baclofen, and hormones | - Heterogeneity of dosages and outcome measures - Scarcity of placebo-controlled RCTs | More rigorous research designs are needed to increase knowledge and further develop vulvodynia treatments |
| Bajzak et al., 2023 | Scoping Review | To review the current evidence on the efficacy of pharmacological treatments for managing LPV | Research studies on pharmacological treatments for LPV | - Limited evidence for the efficacy of pharmacological therapies for LPV. - Descriptive studies show pain reduction with tricyclic antidepressants, milnacipran, injectable anaesthetics, and BT, with mixed outcomes for quality of life and sexual function | Literature restricted to the last decade, potentially excluding older but relevant studies | - Address methodological limitations - Include diverse samples of individuals with LPV in future studies, considering education, race / ethnicity, relationship status, and sexual and gender identities |
| Schlaeger et al., 2023 | Topical Review | To review the presentation, evaluation, and treatments of vulvodynia, to inform clinical decision making | Research studies on treatments for vulvodynia / vestibulodynia / generalised vulvodynia | Treatments with the highest evidence for reducing pain and dyspareunia:   - - multimodal physical therapy   - acupuncture,   - intravaginal TENS   - overnight 5% lidocaine ointment   - oral desipramine with 5% lidocaine cream   - intravaginal diazepam with TENS   - BT type A   - enoxaparin sodium injections   - vaginal dilators   - EMG biofeedback   - Hypnotherapy   - CBT | Lack of rigorous RCTs with large sample sizes | - Conduct research focusing on the aetiology and characterisation of vulvodynia - Conduct more rigorous trials |
| Penteado et al., 2023 | Topical Review | To develop strategies for individualising multidisciplinary therapy for vulvodynia | Clinical trials on vulvodynia treatments and studies on predictive factors, published from 2008 to 2022 | An individualised multidisciplinary therapy model includes psychotherapy, medical care, and physical therapy | - Scarcity of RCTs with substantial sample sizes - Limited analysis of predictors and mediators of treatment outcomes | Conduct more rigorous research with robust samples to identify predictors and mediators of treatment outcomes |
| Karp et al., 2019 | Methodological Review | To describe the authors’ methodology for BT treatment of CPP in women, and place it in the context of the literature on techniques for this use | Research studies on BT and CPP, including vulvodynia / vestibulodynia | - Findings support the safety and tolerability of BT injections in the pelvic floor muscles, with lower doses linked to fewer bowel and bladder side effects - Key methodological factors include needle guidance technique, anatomical landmarks, and injection tolerability in office settings | Lack of complete information in published reports precludes recommendations for a single best methodological approach | - Conduct randomised double-masked placebo-controlled trials to provide detailed efficacy and safety data - Ensure complete reporting of methodological details and efficacy and safety data |
| Starzec-Proserpio et al., 2023 | Scoping Review | To review and summarise the published evidence on the effects of lasers for treating vulvodynia | Research studies on different types of laser treatment for vulvodynia | Limited evidence supports laser therapy for vulvodynia, but conclusions are restricted due to poor-quality evidence | - The limited number and poor quality of existing studies - The broad scope of the included diagnoses related to vulvodynia | Conduct robust RCTs to explore the effectiveness of different laser types |
| Rains et al., 2024 | Scoping Review | To explore the current evidence on the efficacy and effectiveness of multimodal or interdisciplinary interventions for treating LPV | Studies that compared treatments across modalities or applied multimodal / interdisciplinary approaches | Insufficient evidence to recommend a specific treatment or interdisciplinary management strategy for LPV, due to a lack of head-to-head trials and well-designed studies on multimodal interventions | - Limited to contemporary management of LPV - Only detailed interventions that are compared across treatment modalities or that integrate an interdisciplinary approach to LPV management | Design studies with appropriate comparators and blinded assessors to strengthen evidence |
| Leusink et al., 2018 | Systematic Review | To systematically review the literature on the relationship between VVC and PVD | Cohort and case-control studies comparing women with PVD to healthy controls regarding a history of VVC | Women with PVD are more likely to report a history of VVC | - The methodological and clinical heterogeneity of included studies precluded a meta-analysis - Primary studies relied on self-reports of VVC, thereby limiting conclusions | - Conduct large cohort studies of women with VVC to assess PVD incidence, including immunocompromised patients and those without VVC, while adjusting for sexual behaviour - Healthcare providers could consider a diagnosis of PVD in women with self-reported VVC |
| Chisari et al., 2021 | Systematic Review | To identify and evaluate psychosocial factors affecting pain and sexual outcomes in vulvodynia and propose a psychosocial model | Observational / experimental studies reporting on associations between psychosocial factors and pain / sexual outcomes in adult women with PVD | Anxiety, depression, and intimacy factors are linked to pain and sexual functioning in PVD, but limited data prevents the development of a psychosocial model | - The relationships among factors are unclear due to cross-sectional studies - Research is limited to Canada and women aged 18–45, with a lack of psychological theory and generally low-to-medium study quality | Explore psychosocial factors in generalised vulvodynia, considering differences across vulvodynia subsets, and test tailored, theory-based treatments |
| Shallcross et al., 2018 | Systematic Review and Meta-Ethnography | To explore, analyse, and summarise the experiences of women living with vulvodynia | Qualitative studies on vulvodynia / vestibulodynia experience | Psychological difficulties and social challenges are consistently reported | Exclusion of non-English studies and lack of partner perspectives | Include diverse ethnic, cultural, and sexual orientation groups in future research |
| Niedenfuehr et al., 2023 | Scoping Review | To synthesise and analyse emerging literature describing the psychosocial barriers that exist for people with vulvodynia / PVD | Studies on physical, psychological, and interpersonal barriers | Psychosocial and environmental barriers and structural determinants – such as delayed diagnosis, low health literacy, cost, transportation, and racial disparities – adversely affected individuals with vulvodynia | - Studies mainly focused on US/Canada - Limited representation of severe cases - Cross-sectional study designs - Limited discrimination between generalised vulvodynia and PVD | Broaden research scope to include abstracts, dissertations, and other reviews |
| Dagostin Ferraz et al., 2024 | Systematic Review and Meta-Analysis | To assess the presence of anxiety, depression and somatisation in women with vulvodynia | Case-control and cross-sectional studies on vulvodynia and psychological symptoms | Women with vulvodynia had higher anxiety, depression, and somatisation scores when compared to women without vulvodynia | Low methodological quality of the included studies | Improve study designs and address methodological limitations |
| Mocini et al., 2024 | Systematic Review | To review the evidence on the link between weight, nutrition, and metabolic disorders in vulvodynia | Studies on vulvodynia that evaluated nutritional and dietary aspects, body mass index, and metabolic disorders | No conclusive evidence regarding nutritional and metabolic factors influencing vulvodynia onset or treatment | Differences in sample sizes, measurement methods, and the inclusion of studies of fair quality, along with non-standardised terminology for vulvodynia | Collect further data to understand the relationship between vulvodynia and metabolic and nutritional factors |
| Loganathan et al., 2022 | Systematic Review | To evaluate the quality of online medical information about vulvodynia targeted at patients | Web pages related to vulvodynia | Most online vulvodynia resources found through search engines are not clinician-endorsed, as they lack readability and accuracy, raising concerns about their safety and effectiveness | Focused on English-language pages and the first three pages of search results | - Conduct qualitative research with patient groups to understand their perceptions and experiences with online vulvodynia information, including its impact on their interactions with clinicians and healthcare decision-making - Incorporate patient feedback on online information to improve quality and usability |
