# Supplementary material for: A scoping review of vulvodynia research: Diagnosis, treatment, and care experiences
Source: Womens Health (Lond). 2025 Jun 17;21:17455057251345946. doi: 10.1177/17455057251345946 (PMC12174717; doi:10.1177/17455057251345946)
Supplement: sj-docx-4-whe-10.1177_17455057251345946 – Supplemental material for A scoping review of vulvodynia research: Diagnosis, treatment, and care experiences [file sj-docx-4-whe-10.1177_17455057251345946.docx]

| **Appendix C – Data Extraction Tables for RQ1: “What clinical and/or psychosocial research exists on diagnosis and treatment of vulvodynia?”** | | | | | | | |
| --- | --- | --- | --- | --- | --- | --- | --- |
| **Diagnosis of Vulvodynia** | | | | | | | |
| **DIAGNOSTIC MEASURES** | | | | | | | |
| **Study ID** | **Setting** | **Methodology** | **Design** | **Participants** | **Aim** | **Outcomes and Measures** | **Key Findings** |
| Cyr et al., 2016 | Canada | Quantitative | Clinical trial | Women with PVD (N=26) | To investigate the test-retest reliability of the algometer and evaluate its convergent validity for vestibular pain assessment | Vestibular pain was assessed with the new algometer and vulvalgesiometer during two different sessions 2 to 4 weeks apart | The algometer is a reliable and valid instrument for measuring pain threshold and pain pressure tolerance |
| Dargie et al., 2016 | Canada | Quantitative | Clinical trial | Women with CVP (including vulvodynia) (N=288) | To enhance clinical knowledge and apply statistical rigor to create a valid assessment questionnaire—the VPAQ | A set of items created by experts and community members was administered to chronic vulvar pain sufferers, followed by factor analyses to refine the items into scales and subscales | - The VPAQ can be used for assessment, diagnosis, treatment formulation, and treatment monitoring - It could be used to promote communication between patients and providers |
| Dargie et al., 2017 | Canada | Quantitative | Cross-sectional study | Women with CVP (including vulvodynia) (N=182) | To evaluate the VPAQ's psychometric properties and gather user feedback on its utility and accessibility | An online survey of the VPAQ, and feedback about the VPAQ items | - VPAQ subscales demonstrated moderate to strong psychometric properties - The VPAQ was found to be user-friendly by participants |
| Stenson et al., 2021 | USA | Quantitative | RCT | Reproductive-aged women with vestibulodynia (N=16) | To compare the cotton swab test and vulvalgesiometer in assessing vulvar vestibule tenderness in provoked vestibulodynia, and to evaluate topical lidocaine with each method | Vestibular tenderness using light rolling cotton swab tests at 6 sites and the vulvalgesiometer at 2 sites. Lidocaine was applied for 3 minutes, and tests repeated in the initial order | Lidocaine application extinguished mucosal vestibular tenderness detected by both diagnostic tools |
| Vieira-Baptista et al., 2017 | Portugal | Quantitative | Cross-sectional study | Women without vulvodynia (N= 267) | To evaluate the Q-tip Test (QTT) for painful vestibular spots in asymptomatic women | Q-tips were gently pressed at different areas of the vulvar vestibule to obtain a 0–10 score representing the pain felt | - Asymptomatic women can have painful vestibular spots in the absence of vulvodynia - A positive QTT cannot be considered equivalent to vulvodynia |
| Goldstein et al., 2020 | USA | Qualitative | Observational study | Women diagnosed with localized vulvodynia (N=25) | To develop a content-valid PRO measure to assess the symptoms and impacts of localized vulvodynia - The Vulvodynia Experience Questionnaire (VEQ) | Two rounds of in-person interviews | The VEQ is a novel method of collecting information on localized vulvodynia symptoms and impacts that may be suitable for use in clinical trials, contingent on psychometric testing |
| Kaarbø et al., 2021 | Norway | Mixed Methods Study | Clinical trial | Women diagnosed with PVD (N=10) | To evaluate the tampon test as a primary outcome measure for an upcoming RCT for women with PVD | Pain intensity with tampon test and semi-structured interviews | The tampon test may underestimate severity of pain among some women with PVD |
| Telisnor et al., 2022 | USA | Quantitative | Secondary analysis | Women with vulvodynia recruited from a double-blind RCT (Steffen et al., 2020) (N=62) | To examine pain locations marked on general body and genital-specific outlines to provide image-based documentation of vulvar pain | Marked pain on a digital genital-specific outline and a digital general body outline to determine Body Surface Area (BSA) | Findings support concurrent validity of the BSA as a measure of pain location using either genital-specific or general body outlines |
|  | | | | | | | |

| **DIAGNOSTIC SUBGROUPS AND PROFILES** | | | | | | | | | | |
| --- | --- | --- | --- | --- | --- | --- | --- | --- | --- | --- |
| **Study ID** | **Setting** | | **Methodology** | | **Design** | **Participants** | | **Aim** | **Outcomes and Measures** | **Key Findings** |
| Nguyen et al.,  2015a | USA | | Quantitative | | Observational study | Women diagnosed with primary or secondary vulvodynia (N=138) | | To determine whether rates of remission differed among women with primary versus secondary vulvodynia | Vulvodynia remission rates according to medical records | Women with primary vulvodynia are less likely to experience remission compared to those with secondary onset |
| Reed et al., 2016b | USA | | Quantitative | | Longitudinal study | Women with vulvodynia (N=293) | | To define the heterogeneity of vulvodynia by determining data-driven subgroups within the vulvodynia diagnosis | Vulvar pain characteristics and subgroup classifications identified via exploratory cluster analysis | Comorbid pain conditions best separated the groups, while primary vs. secondary vulvodynia did not |
| Basha et al., 2019 | USA | | Quantitative | | Pilot multicenter study | Women diagnosed with LPV (N=33) | | To investigate the somatosensory profile of patients with LPV by comparing thermal and mechanical pain thresholds | Pain characteristics, vestibular cold allodynia, and sensory threshold measured via semi-standardized questionnaire on clinical characteristics and sensory testing for thermal and mechanical thresholds | Vestibular cold allodynia may be a feature of PLV, with reduced forearm pain thresholds suggesting altered sensory processing, though its link to central sensitization is unclear |
| Estibeiro et al., 2022 | USA | | Quantitative | | Case-control retrospective study | Women diagnosed with vulvodynia and controls (N=444) | | To assess menstrual cycle characteristics | Menstrual cycle length, flow, menstrual pain, and tampon use measured via retrospective assessment at three time points (before the study, before vulvodynia onset, and before age 18) | Women with specific premenstrual symptoms also reported higher odds of vulvodynia. Among those with vulvodynia, half reported a change in vulvar pain across the menstrual cycle, with 60% of these reporting greater pain just before and during menstruation |
| Aibel et al., 2023 | USA | | Quantitative | | Retrospective study | Women with CPP, including vulvodynia (N=170) | | To assess whether patterns of pain as marked on a body map of the pelvis exist among common CPP diagnoses and to investigate the association in patients with 1 to 3 CPP diagnoses | Survey data from pelvic pain maps, genitourinary pain and pain catastrophizing | Pelvic body mapping demonstrated that different forms of CPPS displayed different distributions of pain, but mapping was not predictive of any diagnostic group |
| Nguyen et al., 2015b | USA | | Quantitative | | Cross-sectional retrospective study | Hispanic and non-Hispanic Women who reported CVP consistent with vulvodynia (N=1,150) | | To explore whether CVP subtypes differ by ethnicity | Pain characteristics and ethnicity measured via a validated survey | Hispanic women are more likely to report a burning sensation and to have primary vulvodynia compared to non-Hispanic women |
| Saago et al., 2023 | UK | | Quantitative | | Case series | Prepubertal girls experiencing episodes of nocturnal vulval pain and their parents (N=8) | | To explore the clinical features of prepubertal nocturnal vulval pain syndrome and explore possible management and outcomes | Symptoms, clinical features, and management outcomes measured via clinical details from pediatric vulval dermatology clinic database and questionnaire of symptoms administered to parents | Prepubertal nocturnal vulval pain syndrome may be a subset of vulvodynia to be included in the clinical spectrum of night terrors |
|  | | | | | | | | | | |
| **SYMPTOM PRESENTATION/CHARACTERISTICS** | | | | | | | | | | |
| **Study** | **Setting** | | **Methodology** | | **Design** | **Participants** | | **Aim** | **Outcomes and Measures** | **Key Findings** |
| Phillips et al., 2015 | USA | | Quantitative | | Case series | Premenopausal and Postmenopausal women enrolled in a clinical trial for PVD (N=100) | | To determine whether there are differences in the clinical presentation of symptoms and vulvar pain ratings | Questionnaire data on vulvar pain presence, pain characteristics, and women's demographic and reproductive health history | Pre- and postmenopausal women with PVD have similar pain scores and clinical symptoms, except for a higher incidence of burning in postmenopausal women |
| Brown et al., 2015c | USA | | Quantitative | | Cross-sectional study | Women with PVD (N=92) | | To examine the effect of race on symptom reporting pain between black and white women with PVD | Self-reported questionnaire assessing symptom reporting and descriptors of symptoms | Black women are less likely to self-report their vulvar pain as burning, the classic symptom of PVD |
| Tersiguel et al., 2015 | France | | Quantitative | | Clinical trial | Women with vulvodynia (N=16) | | To support the neuropathic component of vulvodynia and assess the presence of anxiety, depression, and other chronic pain conditions in patients | Medical history, cotton-swab test, allodynia assessment, neuropathic pain assessment, assessment of pain and depression and anxiety | There was a presence of a neuropathic component underlying vulvodynia, which was observed in 66% of the patients |
| Aerts et al. 2016 | Canada | | Quantitative | | Cross-sectional study | Clinical and community sample of premenopausal women diagnosed with PVD (N=98) | | To examine the associations among self-reported and objective pain measurements, sexual function, and sexual satisfaction | Self-report pain and sexual function, and objective measurements using the cotton-swab test, vestibular friction procedure, and pressure-pain threshold | Support for the biopsychosocial nature of PVD as women’s pain ratings were not associated with sexual function and satisfaction. |
| Phillips et al., 2016 | USA | | Quantitative | | Randomized double-blind placebo-controlled crossover study | Women diagnosed with PVD (N=92) | | To assess the correlation between non-genital tenderness pain intensity and vaginal algometer pain, and compare scores between women with and without fibromyalgia in PVD | Questionnaires on medical history, assessment of vaginal pain sensitivity, and pain intensity ratings | Women with PVD who experience more severe pain with non-genital tender point palpation also experience more deep vaginal pain on pelvic exam |
| Dargie et al., 2017 | Canada | | Quantitative | | Cross-sectional study | Women with provoked vulvar pain (N=65) | | To explore pain symptoms that resemble NP reported by those with PVD and compare responses with those with an established Neuropathic Pain (NP) condition | Validated measures and additional questions addressed characteristics of vulvodynia and PHN | Women with PVP report some symptoms suggestive of NP characteristics such as evoked pain |
| Haugstad et al., 2018 | Norway | | Quantitative | | Cross-sectional study | Women with PVD (N=30) | | To examine multidimensional parameters including pain, psychological distress and quality of movement to obtain a broader understanding of the somatic and psychological symptoms in PVD | Assessment of pain intensity, psychological distress and quality of movement | PVD women display reduced quality of movement, especially for gait and respiration patterns, increased level of anxiety and high average pain scores |
| Bortolami et al., 2019 | Italy | | Quantitative | | Observational study | Women initiating physical therapy evaluation and treatment for pelvic floor–related dysfunctions (including vulvodynia) (N=85) | | To investigate the associations among pelvic floor function, sexual function, and demographic and clinical characteristics | Female Sexual Function and pain scores | Sexual dysfunction in female patients with pelvic floor muscle dysfunction undergoing physical therapy is significantly correlated with age and high pelvic floor muscle tone |
| Næss et al., 2019 | Norway | | Quantitative | | Exploratory secondary analysis of an assessor blinded comparison study | Women with PVD and asymptomatic controls (N=70) | | To investigate whether there are differences in motor functions, posture and breathing patterns, and differences on how physical health is perceived | Assessment of respiration, posture, and motor function based on the SMT and  physical function and perception of health. Assessment of vaginal resting pressure, PFM strength, and PFM endurance and surface electromyograph | No differences were found between groups in physical functioning, posture, or movement, but women with PVD reported lower general health scores than controls |
| Li et al., 2023 | USA | | Quantitative | | Prospective study | Women seeking care for CPP in a tertiary gynecological pelvic pain clinic (N=200) | | To characterize clinical heterogeneity among women seeking care for CPP in a tertiary multidisciplinary outpatient pelvic pain clinic | Data on pain intensity, interference, catastrophizing, acceptance, pelvic pain syndromes, and psychiatric disorders were compared across social history, treatment, medications, and surgeries | Treatment-seeking women with CPP could be separated into two groups distinguished by pain clusters, pain burden, pain distress and coping, and co-occurring mental health disorders |
| Harlow et al., 2023 | Sweden | | Quantitative | | Retrospective study | Women diagnosed with LPV (N=4,787) | | To test the hypothesis of whether vulvodynia manifests as an altered immune-inflammatory response | Use of Swedish Registry data to capture:  Immunodeficiencies, single organ and multiorgan autoimmune conditions, allergy and atopies, and malignancies involving immune cells across the life course | Women with vulvodynia are substantially more likely to experience a spectrum of immune related conditions across the life course |
| Trutnovsky et al., 2019 | Austria | | Quantitative | | Retrospective study | Women diagnosed with vulvodynia (N=127) | | To explore the symptoms and characteristics of women presenting with vulvodynia and/or chronic pelvic pain to a gynecological outpatient clinic | Review of electronic charts of women diagnosed with vulvodynia or chronic pelvic pain (2010-2015) analyzing pain type, symptom duration, treatments, comorbidities, and patient characteristics | Comorbidities were common, with 40% of women diagnosed with depression, 15% with urological, and 9% with gastrointestinal conditions |
| Dunford et al., 2019 | Australia | | Quantitative | | Retrospective study | Women younger than 18 years who presented with symptoms suggestive of vulvodynia (N=47) | | To describe the presentation, associated symptoms, and management of children and adolescents with vulval pain seeking care at a tertiary pediatric and adolescent gynecology service | Data regarding symptoms, associated symptoms, relevant medical history, clinical findings, and management were extracted from the medical records | Children and adolescents with vulval pain have varied presentations. Many of the pre- and postmenarchal patients had coexisting urinary tract symptomatology |
| Parada et al., 2015 | Canada | | Quantitative | | Descriptive study | Women with clitoral pain (N=126) | | To describe quantitatively the clinical characteristics of clitoral pain, to assess interference with sexual function, and to investigate whether clitoral pain is a unitary category | Assessment of clitoral pain characteristics, short-form McGill pain questionnaire-2, and the female sexual function index | Women with clitoral pain experience significant, distressing, and often long-term pain that affects sexual and daily activities. Two distinct subtypes may exist, each with unique pain characteristics |
|  | | | | | | | | | | |
| **RISK FACTORS AND COMORBIDITIES** | | | | | | | | | | |
| **Study** | **Setting** | **Methodology** | | **Design** | | | **Participants** | **Aim** | **Outcomes (Measures)** | **Key Findings** |
| Reed et al., 2016a | USA | Quantitative | | Longitudinal study | | | Women with vulvodynia (Ν=239) | To estimate the probability of and risk factors for remission, relapse, and persistence among women screening positive for vulvodynia | Survey based assessment of remission without relapse, relapse (after remission), and persistence of a positive vulvodynia screen | Remission of vulvodynia symptoms is common with approximately half of remitters experiencing a relapse within 6-30 months |
| Dargie & Pukall, 2016 | Canada | Quantitative | | Cross-sectional study | | | Women with vulvodynia (N=488) | To explore the sexual and pain histories and pain presentations of women with forms of chronic vulvar pain (i.e., vulvodynia) | Online questionnaire assessing pain characteristics, sexual history, and treatment seeking | Frequent and intense pain is common among people with vulvodynia. Differences on pain development, number of sexual partners, and treatment seeking |
| Reed et al., 2017 | USA | Quantitative | | Longitudinal study | | | Women with vulvodynia (N=2,269) | To assess whether degree of vulvar sensitivity predict vulvodynia characteristics and prognosis | Survey on health status, vulvar symptoms, and validated screenings for vulvodynia, comorbid pain conditions, depression, and PTSD, with follow-ups every six months for three years | Women with vulvodynia have increased vulvar sensitivity, but the range is broad. Sensitivity does not correlate with most pain characteristics or prognosis, indicating a positive swab test is not essential for diagnosis |
| Sun et al., 2019 | USA | Quantitative | | Case-control study | | | Women with and without a diagnosis of vulvodynia (N=437) | To explore whether women with vulvodynia experience adverse urinary symptoms in the absence of urological pain | Assessment of Pelvic Pain and Urinary/Frequency (PUF) Patient Symptoms | Women with vulvodynia are substantially more likely to report voiding dysfunction and symptoms of urgency than women with no history of vulvar pain |
| Reed et al., 2019 | USA | Quantitative | | Longitudinal study | | | Women with vulvodynia (N=1,585) | To evaluate the association between past environmental exposures and the presence of vulvodynia | Vulvodynia status, environmental exposure, and home environment assessed at baseline and 24-month follow-up | A positive association between vulvodynia and the reported history of exposures to a number of household and work-related environmental toxins |
| Klann et al., 2019 | USA | Quantitative | | Explorative study | | | Women diagnosed with vulvodynia and controls (N=434) | To explore whether hygienic behaviors were associated with the onset of vulvodynia | Self-administered questionnaires to assess lifetime history of vulvar pain symptom. Examined five specific types of hygienic behaviors and practices: Wearing tight fitting garments, vulva care and cleaning methods, pubic hair removal, douching, and powdering | Wearing tight-fitting jeans or pants and removing hair from the mons pubis area were associated with increased odds of vulvodynia |
| Gómez et al., 2019 | Spain | Quantitative | | Cross-sectional study | | | Women living in Spain through women's associations, midwives, social networks and websites (N=45) | To study the prevalence, epidemiological characteristics and risk factors associated with vulvodynia | Questionnaire on epidemiological aspects, obstetric and gynecological history, the presence of vulvodynia, associated disorders, duration of vulvodynia symptoms, and comorbidities | The prevalence of vulvodynia in Spain is similar to that found in other countries |
| Graziottin et al., 2020 | Italy | Quantitative | | Cross-sectional study | | | Female patients with chronic vulvar pain (N=1,183) | To investigate the epidemiological characteristics and comorbidities of chronic vulvar pain | Data collected on epidemiological aspects, demographic characteristics, obstetric and gynecological history, the presence and duration of symptoms, associated disorders, details of physical examinations and treatment approaches | The main reason for consultation was superficial dyspareunia. The diagnoses of vestibulodynia and vulvodynia must be considered in patients with chronic vulvar pain |
| Basson et al., 2020 | Canada | Quantitative | | Cross-sectional study | | | Women with PVD and a comparison group of women with low sex drive but no pain during intercourse (N=152) | To document the severity of dysmenorrhea in women with confirmed PVD to further clarify reports of comorbidity | Questionnaires on history and severity of their dysmenorrhea | Women with PVD reported more painful, longer, more debilitating, and persistently painful periods as teenagers than controls |
| Wanczyk-Baszak et al., 2022 | Poland | Quantitative | | Retrospective study | | | Prepubertal girls with vulvodynia (N=54) | To identify specific features, highlight potential triggers and concomitant diseases, outline diagnostic criteria | Medical records of an outpatient clinic, a cohort of 54 with vulvodynia | Vulvodynia should be considered in cases of persistent or recurrent vulvar pain, pruritus, and discomfort, even without visible pathology, and includes various non-specific symptoms |
| Cohen-Sacher et al., 2015 | USA | Quantitative | | Cross-sectional study | | | Individuals with chronic vulvar pruritus and vulvodynia   (N=429) | To compare gynecologic and sexual and physical abuse histories | Self-reported questionnaires on symptoms, general medical and gynecological history, the McGill Pain Questionnaire, and sexuality and sexual relations | Patients with vulvar pruritus and vulvodynia report similar rates of sexual and physical abuse |
| Khandker et al., 2019 | USA | Quantitative | | Cross-sectional study | | | Women with and without vulvodynia (N=185) | To examine rumination as a specific stress response involved in the psychobiological mechanism of vulvodynia | A psychosocial survey with questions specific to early-life traumatic events and rumination | A prolonged cognitive stress response may be a mechanism by which early-life chronic stressors contribute to the onset vulvodynia |
| Chandan et al., 2021 | UK | Quantitative | | Retrospective cohort study | | | Women with a history of domestic abuse (N=67,275) | To explore the association between exposure to domestic abuse in women and the development of syndromes indicating central nervous system sensitization (including vulvodynia) | Using the ‘The Health Improvement Network,’ (UK primary care medical records) | Women exposed to domestic abuse experienced an increased risk of developing vulvodynia among other syndromes |
| Mühlrad et al., 2021 | Sweden | Quantitative | | Cross-sectional study | | | Women born between 1973 and 2001 with and without a diagnosis of PVD/vaginismus  Diagnosed with PVD (N=9,247) | To explore the association between birth-related events and the risk of developing PVD/vaginismus during adulthood | Nationwide registry data were used to estimate the association between health during infancy and the onset of PVD/vaginismus later in life using an event probability model | Adverse health at birth was associated with developing PVD/vaginismus later on in life |
| Blaustein et al., 2024 | USA | Quantitative | | Longitudinal study | | | Women with vulvodynia (N=1,647) | To assess the relationship between childhood sexual abuse (CSA), obesity, and vulvodynia | Surveys assessed health status, diagnoses, risk factors, and screening test outcomes for women with vulvodynia | Obesity and vulvodynia were independently linked to a history of childhood sexual abuse, but obesity did not modify this relationship |
| Morgan et al., 2016 | USA | Quantitative | | Population-based genealogy study | | | Women with vestibulodynia and their female relatives (N=183 probands) | To explore whether there is a genetic predisposition for vestibulodynia in close and distantly related female relatives of women diagnosed with vestibulodynia and those specifically treated by vestibulectomy | The Utah Population Database, relative risk estimation, Genealogical Index of Familiarity statistic, comorbidity analysis, and high-risk pedigrees | Vestibulodynia treated by vestibulectomy has a genetic predisposition |
|  | | | | | | | | | | |

| **Treatment of Vulvodynia** | | | | | | | | | | | | | | | | | | | |
| --- | --- | --- | --- | --- | --- | --- | --- | --- | --- | --- | --- | --- | --- | --- | --- | --- | --- | --- | --- |
| **PSYCHOLOGICAL THERAPIES** | | | | | | | | | | | | | | | | | | | |
| **Study ID** | | **Setting** | | **Methodology** | | **Design** | | **Participants** | | **Aim** | | **Exposure** | | **Outcomes (Measures)** | | **Key Findings** | | | |
| Buhrman et al., 2024 | | Sweden | | Quantitative | | Two-arm RCT | | Women with PVD (N=88) | | To examine if an online delivered ACT with a focus on values-based exposure benefits women with PVD | | Online ACT or waitlist control condition with outcome analyses at 10 weeks and 1-year follow-up | | - Sexual Function (Female Sexual Function Index (FSFI)) - Sexual Distress (Female Sexual Distress Scale (FSDS-R)) - Pain Intensity (Multidimensional Pain Inventory (MPI)) - Anxiety (Generalized Anxiety Disorder 7-item scale (GAD-7)) - Depression (Montgomery–Åsberg Depression Rating Scale (MADRS-S)) - Quality of Life (Brunnsviken Brief Quality of Life Inventory (BBQI)) - Mindfulness (Mindful Attention Awareness Scale (MAAS)) - Pain Acceptance (Chronic Pain Acceptance Questionnaire (CPAQ)) - Committed Action (Committed Action Questionnaire (CAQ-8)) | | - Improvement on sexual functioning, pain, and quality of life post-treatment and at 1 year follow-up - No effect on depression and anxiety post-treatment | | | |
| Maathz et al., 2023 | | Sweden | | Quantitative | | RCT | | Women with PVD (N=47) | | To examine the feasibility and preliminary effectiveness of an online ACT protocol (Buhrman et al., 2013) adapted for PVD | | 6-7-week online ACT or a waitlist control | | - Feasibility (author criteria) - Recruitment rate (%) - Treatment credibility (Treatment Credibility Scale (TCS)) - Completion rate (threshold of 75% of participants completing ≥3 of the 6 treatment modules) - Retention in trial (author criteria) - Pain with Sexual Activity (FSDS-R) - Sexual Functioning (FSFI) - Psychological distress (Hospital Anxiety and Depression Scale (HADS)) - Life satisfaction (Satisfaction with Life Scale (SWLS)) - Relationship satisfaction (revised Dyadic Adjustment Scale (rDAS)) - Pain acceptance (CPAQ) - Pain catastrophizing (Pain Catastrophizing Scale (PCS)) | | - Positive ratings for ACT as a potential treatment - Positive effects on pain acceptance and quality of life and some effects on pain catastrophizing, anxiety, sexual function and relationship adjustment | | | |
| Hess Engström et al., 2022a | | Sweden | | Quantitative | | Multicenter RCT | | Women with PVD (N=99) | | To compare the effects of an internet-based treatment for intercourse pain with no intervention before clinical treatment | | 6-week guided internet-based treatment using ACT principles or usual care | | - Pain during intercourse / tampon test (11-point numeric rating scale (NRS)) - Impact on sexual function (4-point NRS) - Pain acceptance (CPAQ-R) - Pain-related behaviors, e.g., “attempts at intercourse”, “sexual activities besides baseline”, “willingness to perform tampon test” (yes/no) | | - Improved pain during intercourse - Higher pain acceptance | | | |
| Hess Engström et al., 2023 | | Sweden | | Quantitative | | Secondary analysis of data from Hess Engström et al. (2022) | | Women with PVD (N=99) | | To evaluate the cost-effectiveness of a guided internet-based intervention for PVD alongside usual treatment | | 6-week guided internet-based treatment using ACT principles or usual care | | - Healthcare-related costs - Health-related quality of life and quality-adjusted life-years (EuroQol 5 Dimension 3 level (EQ5-D-3L)) - Incremental cost-effectiveness ratio (ICER) - Pain acceptance (CPAQ-R) | | Internet-based ACT as an addition to treatment may reduce healthcare utilization | | | |
| Hess Engström et al., 2022b | | Sweden | | Qualitative | | Semi-structured interviews conducted one month after the intervention | | Women with PVD (N=13) | | To describe women's experiences before, under, and after a guided internet-based intervention for vulvodynia | | 6-week guided internet-based treatment using ACT principles or usual care | | Women's experiences with treatment | | Participants reported positive experiences regarding wellbeing and pain management | | | |
| Engman et al., 2022 | | Sweden | | Quantitative | | Replicated single-case experimental design | | Women with vulvodynia aged 18-45 in a stable sexual relationship with a man (N=5) | | To examine a CBT group treatment with partner involvement in vulvodynia | | 15 sessions, including 10 two-hour group sessions for women and three 1-hour couple sessions, with 3-month and 6-month follow-ups | | - Pain intensity (Genital Pain Rating Questionnaire (GPR)) - Pain Catastrophizing (Vaginal Penetration Cognition Questionnaire (VPCQ)) - Sexual function (Female Sexual Function Index (FSFI)) - Avoidance and endurance behaviour (CHAMP Sexual Coping Scale (CSPCS)) - Treatment satisfaction (11-point NRS) | | Some participants showed improvements in sexual functioning, pain catastrophizing, and avoidance, sustained at follow-ups | | | |
| **ACUPUNCTURE** | | | | | | | | | | | | | | | | | | |  |
| **Study ID** | | **Setting** | | **Methodology** | | **Design / Methods** | | **Participants** | | **Aim** | | **Exposure** | | **Outcomes (Measures)** | | **Key Findings** | | |  |
| Schlaeger et al., 2018 | | USA | | Quantitative | | Feasibility RCT | | Women with vulvodynia (N=6) | | To determine the protocol feasibility of a multiple-needle, multisession pilot study of acupuncture for vulvodynia | | Double-blind placebo control using a 13-  needle, 10-session, twice-weekly, standardized acupuncture  treatment protocol | | - Pain (Short-Form McGill Pain Questionnaire (SF-MPQ)) - Sexual Function (FSFI) | | Feasibility of protocol supported to be used in a double-blind efficacy trial of acupuncture for vulvodynia | | |  |
| Fan et al., 2018 | | USA | | Quantitative | | Protocol for RCT | | Women with generalized vulvodynia PVD (N=51) | | To evaluate two acupuncture strategies for reducing vulvar pain and pain during intercourse, and assess the duration of effects | | Two intervention groups and one control group | | - Pain intensity (11-point NRS) - Pain duration (hours of pain per day at baseline) - Pain during intercourse (11-point NRS) - Pain intensity prior to and during cotton swab test (11-point NRS) | | N/A | | |  |
| Steffen et al., 2020 | | USA | | Quantitative | | RCT | | Women with generalised or PVD and acupuncturists (N=48) | | To describe a systematic approach to implement and measure blinding success in a double-blind phase 2 RCT testing the efficacy of acupuncture for the treatment of vulvodynia | | Randomised 1:1 to either penetrating or placebo needles, receive a 13-needle, twice-weekly, 10-session, 5-week acupuncture treatment protocol | | - Blinding success (author criteria) | | This approach can potentially improve the understanding of unblinding and assist in developing rigorous trials | | |  |
| Desloge et al., 2023 | | USA | | Qualitative | | Open-ended responses to a vulvodynia module of PAIN*Report*It | | Women with vulvodynia (N=50) | | To explore motivations of women participating in a double-blind RCT of acupuncture for vulvodynia | | Asked to respond to one question: ‘Tell me about why you decided to participate in this study’ in free text boxes | | Patterns in motivation for study participation | | - Motivations included reducing pain and healthcare costs - Responses indicate that acceptability of acupuncture as treatment | | |  |
| **PHYSICAL THERAPY** | | | | | | | | | | | | | | | | | | |  |
| **Study ID** | | **Setting** | | **Methodology** | | **Design / Methods** | | **Participants** | | **Aim** | | **Exposure** | | **Outcomes (Measures)** | | **Key Findings** |  | |  |
| Belanger et al., 2022 | | Canada | | Quantitative | | Observational study | | Women with PVD (N=105) | | To explore whether pre-treatment pain characteristics, psychological variables, and PFM function predict the response to physical therapy | | 10 weekly sessions of individual PT comprising education, PFM exercises with biofeedback, manual therapy, and dilators with treatment outcomes evaluated at pre-treatment, post-treatment, and 6-month follow-up. | | - Pain intensity (11-point NRS) - Sexual function (FSFI) | | PVD secondary subtype, lower PFM tone, and fear of pain were predictors of treatment response |  | |  |
| Varela et al., 2017 | | USA | | Quantitative | | Case study | | A 53-year-old female patient with vestibulodynia | | To present the results of use of BurstDR spinal cord stimulation (SCS) in the treatment of vestibulodynia | | A 2-week SCS trial followed by implantation of a permanent SCS stimulator | | - Pain (Visual Analogue Scale (VAS)) | | Patient reported reduction in vulvar pain at the end of the trial without any adverse effects |  | |  |
| Murina et al., 2023 | | Italy | | Quantitative | | Single-blind RCT | | Women with PVD (N=78) | | To evaluate the effects of the combination of frequency and pulse duration in reducing pain intensity and dyspareunia and the change in symptom patterns relative to the number of TENS applications | | TENS vs. control | | - Mean change of burning/pain and dyspareunia (VAS at baseline, 60, and 120 days) - Pain (Vulvar Pain Functional Questionnaire (V-Q)) - Sexual Function (FSFI) - PFM activity (vaginal surface electromyography) | | TENS group reported a reduction in pain and dyspareunia relative to control |  | |  |
| Peper et al., 2015 | | USA | | Qualitative | | Case study | | 23-year-old woman with vulvodynia | | To describe the process of a holistic biofeedback-based intervention | | r-session treatment intervention teaching diaphragmatic breathing to transform shallow thoracic breathing into slower diaphragmatic breathing | | Self-report on symptoms and sexual function | | Patient reported alleviation of symptoms and improved sexual function |  | |  |
| Kaarbø et al., 2022 | | Norway | | Mixed Methods | | Feasibility study with a single-arm before-after trial and qualitative interviews | | Women with PVD (N=10) | | To assess the feasibility, implementation, acceptability, and potential effectiveness of SCT in a full-scale RCT | | Self-report questionnaires, tampon test, and 14-day diary of emotional states | | - Recruitment rate (%) - Response rate (%) - Adherence (n) - Utility (based on tampon test data and participants’ experiences with the test) - Adverse events (n) - Acceptability (6-point Global Perceived Effect Scale (GPE)) | | SCT was an acceptable treatment and participants adhered to questionnaires and tampon test, but adherence to the diary was poor |  | |  |
| Danielsen et al., 2022 | | Norway | | Qualitative | | Case study | | A patient with PVD and a physiotherapy student | | To explore the content of an SCT session using a body-mind approach, led by a physiotherapy student in an outpatient clinic | | Video-based case study of the student-patient encounter midway through an SCT treatment course | | Content Analysis of the video | | Empathic relationships with the patient and communicative aspects during treatment were identified as important during treatment |  | |  |
| Danielsen et al., 2024 | | Norway | | Qualitative | | Semi-structured interviews towards the end of the treatment period, and follow-up interviews one year later | | Women with PVD (N=10) | | To explore participants’ experiences with SCT over time | | SCT intervention for PVD | | Experiences following SCT intervention | | SCT can initiate and support several beneficial processes that promote embodiment and sexual health among women with PVD |  | |  |
| **PHARMACOLOGICAL TREATMENTS (MEDICATION, TOPICAL TREATMENTS, AND INJECTIONS)** | | | | | | | | | | | | | | | | | |  |  |
| **Study ID** | | **Setting** | | **Methodology** | | **Design / Methods** | | **Participants** | | **Aim** | | **Exposure** | | **Outcomes (Measures)** | | **Key Findings** | |  |  |
| Ruoss et al., 2021 | | Australia | | Quantitative | | Observational, retrospective clinical audit | | Women who were prescribed AOO (amitriptyline 0.5% and oestriol 0.03% in organogel) (N=376) | | To examine the efficacy of AOO as treatment for vulval pain, including vulvodynia | | AOO | | - Treatment effectiveness (Pelvic Pain Impact Questionnaire) | | Topical AOO was effective and well-tolerated for treatment of vulval pain | |  |  |
| Ofir et al., 2021 | | Israel | | Quantitative | | Transversal, monocentric, blinded, observational study | | Women with severe PVD (N=31) | | To assess the long-term beneficial effects of enoxaparin on PVD | | Enoxaparin | | - Treatment satisfaction (Global Impression of Improvement (PGI-I)) - Pain (11-point NRS) - Frequency of intercourse (number of times per month) | | Positive effects of enoxaparin three years post-treatment, with improved pain during intercourse | |  |  |
| Nacthigall et al., 2022 | | USA | | Quantitative | | RCT, double-blind, placebo-controlled | | Postmenopausal sexually active women with secondary PVD (N=32) | | To determine the efficacy of low-dose topical sinecatechins ointment in reducing PVD | | Topical sinecatechins ointment | | - Vaginal pH - Vaginal maturation index | | Topical sinecatechins ointment is a novel therapeutic alternative for pain reduction and improved sexual function | |  |  |
| Brown et al., 2018b | | USA | | Quantitative | | Secondary analysis of a multicenter, randomized, placebo-controlled trial (Brown et al., 2018a) | | Women with PVD (N=89) | | To explore racial differences among participants in a trial of gabapentin | | Gabapentin | | - Pain intensity (Tampon Test, 11-point NRS) | | Pharmacologic pain interventions may vary by demographics, with white women showing better response to gabapentin than black women | |  |  |
| Villa-Muñoz et al., 2023 | | Spain | | Quantitative | | Open-label exploratory study | | Women with vestibulodynia  (N=35) | | To assess the effect of BoNT/A injections into the vulvar vestibule and implement a protocol for diagnosing vestibulodynia to optimise treatment | | BoNT/A injections with outcomes assessed at 8-, 12- and 24-weeks post-treatment | | - Day-to-day pain (VAS) - Pain (cotton swab test, VAS) - Sexual functioning (FSFI; Marinoff’s Dyspareunia Scale) - Emotional functioning (Hospital Anxiety and Depression Scale) - Pain catastrophising (PCS) - Vulva trophism and tenderness - PFM electrical activity (surface electromyography) | | - BoNT/A injections showed beneficial effects for treating vestibulodynia - Highlighted the importance of adapting treatment to clinical presentation and patient history | |  |  |
| Bhuiyan et al., 2023 | | USA | | Quantitative | | Observational, retrospective case series | | Adolescents with vulvodynia refractory to multimodal management  (N=3) | | To assess the effectiveness of transvaginal BoNT injection into the pelvic floor in select adolescents | | Transvaginal injections of BoNT into the pelvic floor | | Response to BoNT treatment on vulvar pain symptoms (self-report) | | Transvaginal BoNT injection into the pelvic floor can be an effective treatment for adolescents with vulvodynia | |  |  |
| **LASER THERAPY** | | | | | | | | | | | | | | | | | |  |  |
| **Study ID** | | **Setting** | | **Methodology** | | **Design / Methods** | | **Participants** | | **Aim** | | **Exposure** | | **Outcomes (Measures)** | | **Key Findings** | |  |  |
| Goldstein et al., 2019 | | USA | | Quantitative | | Three-site, prospective, double-blind, sham-controlled investigator-initiated study | | Women with vestibulodynia (N=53) | | To examine the safety and efficacy of CO2 fractional laser therapy for treating vestibulodynia | | Randomized to receive active or sham treatment 2:1 | | - Pain (cotton swab test) - Sexual distress (FSDS-R) - Urinary and pain symptoms (O’Leary/Sant Voiding and Pain Indices (ICSI/ICPI)) | | CO2 fractional laser therapy improved subjective measures of pain and pain related to sexual activity | |  |  |
| Morin et al., 2022 | | Canada | | Quantitative | | Randomized prospective pilot study | | Women with PVD (N=40) | | To evaluate the feasibility and acceptability of HILT and explore the effects of active and sham HILT on pain intensity during intercourse | | Bi-weekly sessions of active or sham HILT for six weeks | | - Improvement of pain during intercourse (yes/no) - Perceived improvement and sexual satisfaction (11-point NRS) | | - HILT was feasible and acceptable - Improvements in pain and sexual function were observed | |  |  |
| **OTHER TREATMENTS** | | | | | | | | | | | | | | | | | |  |  |
| **Study ID** | | **Setting** | | **Methodology** | | **Design / Methods** | | **Population** | | **Aim** | | **Exposure** | | **Outcomes (Measures)** | | **Key Findings** | |  |  |
| Novak-Hlebar et al., 2022 | | Croatia | | Quantitative | | Case study | | 53-year-old postmenopausal woman with vulvodynia | | To examine the effect of multidisciplinary treatment | | A multidisciplinary treatment  approach involving an anaesthesiologist, gynaecologist, urologist, psychiatrist, and dermatologist | | - Vulva pain (Self-reported) - Quality of life (Dermatology Life Quality Index (DLQI)) - Anxiety (State-trait Anxiety Inventory (STAI)) - Depression (Beck Depression Inventory II) | | Targeted  psychiatric treatment together with acupuncture treatments and support by a gynaecologist improved symptoms and quality of life | |  |  |
| Das et al., 2020 | | USA | | Mixed methods | | Cohort study | | Patients with vulvodynia who underwent modified vestibulectomy (N=22) | | To describe patient outcomes after modified vestibulectomy for vulvodynia | | Modified vestibulectomy | | - Pain change/improvement (Semi-structured interviews) - Satisfaction (Semi-structured interviews) - Sexual function (Semi-structured interviews) | | - Improved pain and high overall satisfaction post-surgery - Variability observed in sexual function improvement | |  |  |
| Giovanis & Zeszutek, 2020 | | USA | | Quantitative | | Case study | | 40-year-old woman with vulvodynia | | To present a case of vulvodynia managed with osteopathic manipulative treatment | | Osteopathic manipulative treatment using balanced ligamentous tension, myofascial release, and osteopathy in the cranial field | | Self-reported symptoms of pain and depression | | Osteopathic manipulative treatment was effective in managing vulvodynia symptoms | |  |  |
| Carey et al., 2022 | | USA | | Quantitative | | Multicenter randomized controlled trial rationale and design | | Premenopausal and perimenopausal women with vestibulodynia | | To evaluate the efficacy of peripherally and centrally acting medications currently used in clinical practice for treating unique vestibulodynia subtypes | | Treatment conditions: peripheral 5% lidocaine + 0.02% oestradiol cream + oral placebo, central nortriptyline + placebo cream, combined treatments, or placebo for both | | - Pain (Tampon test & McGill Pain Questionnaire) - Perceived physical, mental and sexual health (standardised questionnaire) - Cytokines and microRNAs in local vaginal and circulating blood samples (multiplex assays and RNA sequencing) | | N/A | |  |  |
| Cutting et al., 2023 | | USA | | Quantitative | | Multi-site randomized, double-blind trial | | Premenopausal and perimenopausal women with vestibulodynia (N=78) | | To describe the design and present baseline participant characteristics of our ongoing RCT for vestibulodynia (Carey et al., 2022) | | Treatment conditions: peripheral 5% lidocaine + 0.02% oestradiol cream + oral placebo, central nortriptyline + placebo cream, combined treatments, or placebo for both | | Assessment of participant characteristics and vulvodynia subtypes (demographic questionnaire) | | N/A | |  |  |
