# Supplementary material for: A scoping review of vulvodynia research: Diagnosis, treatment, and care experiences
Source: Womens Health (Lond). 2025 Jun 17;21:17455057251345946. doi: 10.1177/17455057251345946 (PMC12174717; doi:10.1177/17455057251345946)
Supplement: sj-docx-5-whe-10.1177_17455057251345946 – Supplemental material for A scoping review of vulvodynia research: Diagnosis, treatment, and care experiences [file sj-docx-5-whe-10.1177_17455057251345946.docx]

| **Appendix D – Data Extraction Tables for RQ2: “What clinical and/or psychosocial research exists on patient experience of vulvodynia?”** | | | | | | | | |
| --- | --- | --- | --- | --- | --- | --- | --- | --- |
| **Partner** | | | | | | | | |
| **Study ID** | **Setting** | **Methodology** | **Design** | **Participants** | **Aim** | **Exposure** | **Outcomes (Measures)** | **Key Findings** |
| Davis et al., 2015 | Canada | Quantitative | Cross-sectional | Women with PVD and their partners (N= 354) | To examine if partner pain-related cognitive variables are linked to patient pain and depression, and whether partner behavioral responses mediate this effect | N/A | Questionnaires answered individually by women and their partners assessing partner pain catastrophizing and attributions, patient pain and depression | Partner behavioral responses to pain are associated with pain and depressive symptoms. It may be important to involve partner in PVD therapy |
| Rosen et al., 2015a | Canada | Quantitative | Dyadic daily experience study | Women diagnosed with PVD and their cohabitating male partners (N=69) | To investigate the within‐person associations between partner responses to painful intercourse and the sexual and relationship satisfaction of affected couples | 8‐week Internet‐based daily experience study | Measures: Partner responses, sexual relationship and satisfaction | Facilitative partner responses increase sexual and relationship satisfaction compared to solicitous partner responses |
| Rosen et al., 2015b | Canada | Quantitative | Dyadic daily experience study | Women diagnosed with PVD and their cohabitating male partners (N=69) | To examine male partner bias and accuracy in estimating women's pain and how men's relationship satisfaction affects this estimation | 8-week Internet based daily diary study | Pain intensity perceptions of males and pain intensity of females, and relationship satisfaction | Men underestimate women's pain, hindering their emotional and behavioral responses, which may negatively impact coping with vulvodynia |
| Rosen et al., 2015c | Canada | Quantitative | Dyadic daily experience study | Women diagnosed with PVD and their cohabitating male partners (N=69) | To explore partner responses to pain and the impact on pain and anxiety | 8‐week Internet‐based daily experience study | Sexual functioning, sexual and relationship satisfaction, anxiety and depression | Focusing on partner responses may enhance the quality and efficacy of interventions aimed at reducing pain in women with vulvodynia and couples' psychological distress |
| Rosen et al., 2016 | Canada | Quantitative | Secondary analysis of data from Bois et al., 2015 | Women diagnosed with PVD and their partners  (N=50) | To explore how disclosure, empathic response, and relationship adjustment relate to women's pain during intercourse and quality of life | N/A | Self-report measures of perceived empathic response and disclosure. Observational measures of empathic response and disclosure | Disclosure and empathic response may help women sustain the quality of their lives, and couples maintain the quality of their overall relationship |
| Rosen et al., 2017 | Canada | Quantitative | Cross-sectional | Women with PVD and their partners (N=50) and control couples (N=50) | To compare the IEMSS components (i.e., balance of sexual rewards and costs, balance of relative sexual rewards and costs, equality of sexual costs between partners) and the sexual satisfaction of couples | N/A | Standardized measures of sexual exchange and sexual satisfaction | Lower IEMSS exchange components contribute to overall lower satisfaction in couples affected by PVD |
| Rancourt et al., 2017 | Canada | Quantitative | Cross-sectional | Women diagnosed with PVD and their partners  (N=87) | To examine associations between collaborative and negative sexual communication patterns (SCPs) and women's pain and the sexual and relationship adjustment of women with PVD and their partners | N/A | Standardized measurements of SCPs, Pain, Sexual function satisfaction, sexual distress, and sexual satisfaction | Collaborative SCP may benefit couples' sexual and relational well-being, whereas negative SCP may impede sexual and relational adjustment to PVD |
| McNicoll et al.,  2017 | Canada | Quantitative | Cross-sectional | Women experiencing pain symptoms consistent with PVD  (N=140) | To examine whether women’s sexual assertiveness mediated the association between women’s perception of facilitative partner responses and women’s sexual function and satisfaction | N/A | Questionnaires measuring sexual assertiveness, sexual function and satisfaction and partner response to pain | Women's higher sexual assertiveness mediated the association between their greater perceived facilitative partner responses and their improved sexual function and satisfaction |
| Sadownik et al., 2017 | Canada | Qualitative | Cross-sectional | Male intimate partners of women with PVD who completed the Multidisciplinary Vulvodynia Program (MVP) (N=16) | To explore the experiences of male partners of women with dyspareunia, secondary to PVD, who participated in the MVP | Multidisciplinary Vulvodynia Program (MVP) | Online or telephone semi structured interviews | PVD caused strain and communication challenges, but also opportunities for growth. Participants in the treatment program reported improvements in knowledge, communication, and psychological and sexual health |
| Gauvin et al., 2019 | Canada | Quantitative | Cross-sectional | Women diagnosed with PVD and their male partners (N=8) | To assess the quality of interactions that occur between women with PVD and their partners when discussing a moderately distressing topic not specific to their experience with PVD |  | Measures of Dyadic adjustment scale, Golombok-rust inventory of sexual satisfaction, Rapid marital interaction coding system | Empathy may help to buffer the effects of PVD on relationship satisfaction, whereas some types of self-disclosure may exacerbate the sexual impact of PVD |
| Bosisio et al., 2020 | Canada | Quantitative | Used data from previous studies: Bois et al., 2016 and Rosen et al., 2016 | Couples coping with PVD (N=50) | To examine the associations between depressive symptoms, attachment, and perceived and observed partner responsiveness |  | Videotaped discussion and self-report measures of depressive symptoms, attachment, and perceived partner responsiveness | Couple therapy focusing on depressive symptoms and relationship insecurity could increase responsiveness in couples coping with PVD |
| Charbonneau-Lefebvre et al., 2021 | Canada | Quantitative | Cross-sectional | Couples coping with PVD (including three same sex couples)  (N=125) | To examine the mediating role of facilitative and negative partner responses in the associations between attachment and relationship and sexual adjustment |  | Self-report questionnaires on attachment, partner responses, sexual satisfaction and distress, and relationship satisfaction | Partner’s and women’s women attachment orientation may help clinicians better understand couples’ coping with PVD |
| Bennett-Brown et al., 2022 | USA | Quantitative | Descriptive Study | Individuals of color who experience chronic vulvovaginal pain (N=333) | To explore partner’s supportiveness, other sources of emotional support, and their satisfaction and stress surrounding sexual activity |  | Questionnaires on partner support, dissatisfaction and distress about sex life, emotional support resources | Partner supportiveness was associated with less distress and less dissatisfaction surrounding sexual activity |
| Schneider et al., 2022 | Norway | Qualitative | Cross-sectional | Women with PVD (N=9) | To explore the experiences of sexual intimacy in women living with PVD | N/A | In-depth interviews | Women’s tendency to endure painful intercourse and not tell the partner is driven by fear of rejection and conflict |
| Santerre-Baillargeon et al., 2023 | Canada | Quantitative | RCT | Couples coping with PVD  (N=108) | To examine women's and partners' pain self-efficacy and pain catastrophizing as mediators of change in CBCT, using topical lidocaine as a control group |  | Assessed at pre-treatment, post-treatment, and six-month follow-up. Measures of pain catastrophizing, self-efficacy, sexual function and distress | Pain catastrophizing may be a mediator specific to CBCT for PVD |
| Myrtveit-Stensrud et al., 2023 | Norway | Qualitative | Cross-sectional | Women diagnosed with vulvodynia and their partners (N=8) | To explore how heterosexual couples experience living with vulvodynia |  | Semi-structured interviews | Couples struggle with understanding the pain, as well as navigating their social and sexual lives |
| Ekholm et al., 2023 | Sweden | Quantitative | Observational Study | Couples coping with vulvodynia (N=62) | To explore sexual communication patterns, including communication quality, sexual assertiveness, self-disclosure, and their associations with pain intensity | N/A | Self-report questionnaire data and behavioral observational data in the form of video-recorded conversations | There is a need to direct treatment interventions toward couples' sexual communication quality (i.e., levels of validation and invalidation) |
| **Pregnancy** | | | | | | | | |
| **Study ID** | **Setting** | **Methodology** | **Design** | **Participants** | **Aim** | **Exposure** | **Outcomes (Measures)** | **Key Findings** |
| Möller et al., 2015 | Sweden | Quantitative | Retrospective, population-based register study | All women born in Sweden 1973–83 who gave birth for the first time or remained nulliparous during the years 2001–09 (N= 454,913) | To compare sociodemographic, parity and mode of delivery between women diagnosed with vaginismus or LPV to women without a diagnosis before first pregnancy | N/A | Nationally linked registries were used to identify the study population | Women with vaginismus/LPV are less likely to give birth and more likely to opt for caesarean sections. Both sexual and reproductive function should be addressed |
| Smith et al., 2018 | Canada | Quantitative | Cross-sectional | Maternity care providers  (N=116) | To examine maternity providers' recommendations for pregnant women with vulvodynia management during labor | N/A | Questionnaires regarding pregnancy and childbirth care in women with vulvodynia | Need for increased education of vulvodynia aimed at providers of antenatal, labor, and postnatal care |
| Smith et al., 2022 | Canada | Quantitative | Prospective Case-Control study | Pregnant individuals diagnosed with vulvodynia (N=57) and control individuals (N=41) | To assess changes in vulvar pain from pregnancy to postpartum and compare pain anxiety, fear of childbirth, depression, and anxiety symptoms between groups | N/A | Online survey during pregnancy and three and six months postpartum | Pregnant women with vulvodynia experienced postpartum improvements in vulvar pain symptoms. Mode of birth may play a role in symptom trajectory |
| Baril et al., 2023 | USA | Quantitative | Retrospective cohort study | Women with vulvodynia or vaginismus  (N=879) | To evaluate the associations between vulvodynia and vaginismus and obstetric outcomes |  | All birth-related admissions (1999 - 2015) extracted from the Healthcare Cost and Utilisation Project-National Inpatient Sample | Vulvodynia and vaginismus in pregnancy appears underreported in pregnancy compared to reported population rates |
| **Psychosocial Factors** | | | | | | | | |
| **Study ID** | **Setting** | **Methodology** | **Design** | **Participants** | **Aim** | **Exposure** | **Outcomes (Measures)** | **Key Findings** |
| Chisari & Chilcot, 2017 | Italy | Quantitative | Cross-sectional | Women with vulvodynia  (N=335) | To examine whether distress, fatigue, illness perceptions, and cognitive-behavioral factors would be associated with pain severity and interference | N/A | Questionnaires of self-reported pain severity and pain interference | Distress, illness perceptions, fatigue, and cognitive-behavioral are associated with pain severity and interference highlighting the importance of adopting a biopsychosocial approach in this setting |
| Gerhant et al., 2017 | Poland | Quantitative | Case study | A 45-year-old woman with relapse of vulvar pain | To describe the case of vulvodynia coexisting with symptoms of a depressive syndrome |  | Referral to a psychiatry clinic due to the relapse of vulvar pain persisting for half a year and the coexisting symptoms of depression | Vulvodynia often coexists with depression and other functional pain syndromes, increasing the risk of suicide, particularly with depressive symptoms |
| Chisari et al., 2021 | UK | Quantitative | Cross-sectional | Individuals with provoked and spontaneous/mixed vulvodynia (N=349) | Network analysis to explore relations between psychological flexibility, newly emerging relevant psychosocial variables and their associations with vulvodynia outcomes |  | Self-report questionnaires on pain and sexual outcomes, depression, facets of psychological flexibility, body-exposure anxiety during intercourse, unmitigated sexual communion, and perceived injustice | Perceived injustice, pain acceptance, depression, and psychological flexibility appear to be important in vulvodynia |
| Chisari et al., 2022 | UK | Quantitative | Longitudinal Study | Women diagnosed with vulvodynia (N=349) | To explore whether psychological flexibility, body-exposure anxiety and avoidance during sexual activities and perceived injustice assessed at baseline predict pain severity, pain interference, sexual functioning and satisfaction and depression 3 months later |  | Questionnaires on disease severity variables, psychosocial measures, pain severity, interference, sexual and emotional functioning, and sexual satisfaction. 3-month assessment included questions about COVID-related experiences and impact | Pain acceptance and committed action are prospectively associated with pain interference and depression |
| Maunder et al., 2022 | Canada, USA, Australia | Quantitative | Cross-sectional study | Individuals with PVD symptoms  (N=65) | To explore whether pain anxiety, stress, and solicitous partner responses moderated the relationship between penetrative pain and pain-related sexual disability |  | Validate questionnaires assessing pain-related sexual disability, pain anxiety, perceived stress, perceived solicitous partner responding, and level of penetrative pain | High pain anxiety and frequent solicitous partner responses to an individual's pain predicted higher pain-related sexual disability |
| Andersson et al., 2023 | Sweden | Quantitative | Cross-sectional | Patients diagnosed with LPV (N=30) | To identify the prevalence of perfectionism, self-compassion, impostor phenomenon, anxiety, and stress in patients with LPV |  | Questionnaires either at the clinic in paper format or at home in a digital form | Impostor phenomenon and perfectionism were particularly common |
| **Experience of Vulvodynia** | | | | | | | | |
| **Study ID** | **Setting** | **Methodology** | **Design** | **Participants** | **Aim** | **Exposure** | **Outcomes (Measures)** | **Key Findings** |
| Groven et al.,  2016 | Norway | Qualitative | Cross-sectional | Women with vestibulodynia (N=8) | To explore the experiences of Norwegian women living with vestibulodynia | N/A | In-depth interviews | Difficult and ongoing process to fulfil partners’ sexual desires and their own |
| McCann, 2016 | USA | Qualitative | Dissertation | Heterosexual women diagnosed with vulvodynia (N=4) | To develop greater understanding and insight as to the ways in which the lived experience of vulvodynia impacted four heterosexual women's gender identity | N/A | Semi-structured interviews | Both negative and positive impact on gender identity such as isolation, feeling dismissed but also self-advocacy and body appreciation |
| Schlaeger et al., 2019 | USA | Mixed-methods | Cross-sectional | Women diagnosed with vulvodynia  (N=60) | To describe pain experiences and pain relief strategies of women with vulvodynia | N/A | Pain measures, prescribed treatments, and self-reported behaviors with women's free responses | Severe pain in women with vulvodynia may be a clinical indicator of those at higher risk of combining prescription pain medications with alcohol |
| Hintz, 2019 | USA | Qualitative | Cross-sectional | Women with vulvodynia (N=26) | To apply the critical interpersonal and family communication (CIFC) framework within the context of vulvodynia | N/A | Semi-structured interviews | Loss of relational power and problematic media depictions establishing standards for “normal” heterosexual sex |
