# Supplementary material for: A scoping review of vulvodynia research: Diagnosis, treatment, and care experiences
Source: Womens Health (Lond). 2025 Jun 17;21:17455057251345946. doi: 10.1177/17455057251345946 (PMC12174717; doi:10.1177/17455057251345946)
Supplement: sj-docx-6-whe-10.1177_17455057251345946 – Supplemental material for A scoping review of vulvodynia research: Diagnosis, treatment, and care experiences [file sj-docx-6-whe-10.1177_17455057251345946.docx]

| **Appendix E – Data Extraction Tables for RQ3: “What clinical and/or psychosocial research exists on seeking/accessing/receiving care for vulvodynia?”** | | | | | | | | |
| --- | --- | --- | --- | --- | --- | --- | --- | --- |
| **HEALTHCARE ACCESS/EXPERIENCES/BEHAVIOURS** | | | | | | | | |
| **Study ID** | **Setting** | **Methodology** | **Design** | **Participants** | **Aim** | **Exposure** | **Outcome/Measures** | **Key Findings** |
| Adams et al., 2023 | USA | Quantitative | Cross-sectional | Women of color with chronic vulvovaginal pain (N=488) | To explore women’s journey to diagnosis and   perceptions of race-based medical mistrust in pursuit of the  following research questions | N/A | An online survey using the Group-Based Medical Mistrust Scale   (GBMMS) to measure perceptions of trust and support   among healthcare users | Racial and ethnic differences in medical suspicion scores: non-Black Hispanic/Latinx women had the highest, and non-Hispanic/Latinx Black women had the lowest scores |
| Baynes, 2018 | USA | Qualitative | Dissertation | Clinical counsellors, clinical social workers, clinical psychologists,  and marriage family therapists who had treated women with vulvodynia (N=10) | To explore the professional experiences and  practices of therapists who treated women with vulvodynia  and the challenges mental health professionals faced | N/A | Individual interviews with participants | Individual and couples therapy with sensate focus, CBT, and relaxation; therapists unaware of evidence-based protocols, and patient anxiety and depression hinder treatment |
| Bond et al., 2022 | USA | Quantitative | Population-based survey | Women with chronic vulval pain for more than three months (N= 30,676) | To assess care seeking for chronic vulval pain | N/A | Survey asking women if they had ever sought care for their chronic vulvar condition, also evaluating demographic characteristics and comorbidities | Care-seeking behavior varies by pain type: fewer women with contact pain sought care, and those who did consulted fewer providers than women with burning pain. |
| Boyer et al., 2017 | Canada | Quantitative | Cross-sectional | Residents in Canadian Obstetrics and Gynecology (OBGYN) and Family Medicine (FM)  Programs  (N=99) | To assess if post-graduate trainees have more positive attitudes toward women with vulvovaginal pain with an identifiable cause and to examine predictors of comfort in treating vulvodynia | N/A | Online survey related  to demographics, sexuality-related training, sexual attitudes, and comfort treatment and attitudes toward  vulvovaginal pain and its symptoms | More positive attitudes toward patients with visible pathology versus no identifiable cause for vulvovaginal pain, regardless of medical specialty |
| Boyer, 2021 | Canada | Quantitative | Dissertation | Women with pelvic and  vulvovaginal pain (vulvodynia) and medical   residents in Family Medicine and Obstetrics and Gynecology (N=99) | To investigate the healthcare experiences of women with chronic   vulvovaginal pain, vulvodynia | N/A | Three online studies: 1. Analyzed pelvic exam experiences in women with and without chronic intercourse pain, 2. Studied pain and adjustment in women with vulvovaginal pain, 3. Evaluated knowledge, attitudes, and comfort of Canadian medical residents in Family Medicine and Obstetrics/Gynecology | Comfort discussing sexuality in medical practice was a significant predictor of knowledge, attitudes, and comfort with vulvodynia |
| Fernandes et al., 2020 | Norway | Qualitative | Descriptive Study | Gynecologists and general practitioners (N=13) | To understand how Norwegian doctors perceive   female infertility diseases, namely those that are more difficult to diagnose and to treat, and that  are more common in their practice, including vulvodynia | N/A | Telephone or face-to-face interviews with participants | Perceptions of stigmatization, disturbances in women's daily life, diagnostic delay, and need for governmental support |
| Hintz, 2023 | USA | Mixed-methods | Cross-sectional | Women diagnosed with vulvodynia (N=34) | To offer guidance to clinicians counselling patients with vulvodynia by explicating the frequency and difficulty of various salient conversational topics | N/A | Survey indicating the frequency and difficulty of conversational topics and follow-up in-depth interviews | Topics such as sex were rated as among the least difficult to discuss Most participants reported experiencing the facilitative partner response type, which promotes adaptive coping |
| Leusink et al., 2018 | The Netherlands | Qualitative | Cross-sectional | General Practitioners (N=17) | To recognize barriers and facilitators of GPs in the diagnostic process of women presenting with recurrent vulvovaginal complaints | N/A | Focus Group Interviews | Barriers to diagnosing and managing PVD include reluctance to discuss sexual complaints, male gender, emotional responses to uncertainty, and lack of education. Facilitators include female gender and recognizing the benefits of addressing sexual health issues |
| Leusink et al., 2019 | The Netherlands | Qualitative | Cross-sectional | Women with PVD (N=12) | To gain insight into how women with PVD perceive and evaluate condition management by their GP, in order to support GPs in the consultation of women with PVD | N/A | Face-to-face in-depth interviews with participants | Women with PVD prefer a patient-centered approach and want GPs to acknowledge their autonomy and to address sexuality proactively |
| Lountzi & Durand, 2024 | UK, Australia, USA | Qualitative | Cross-sectional | Women with vulvodynia (N=10) | To explore the role of partner support in help-seeking behaviors and pain experiences | N/A | Semi-structured interviews with participants | A lack of continuous care and a multidisciplinary approach, with negative help-seeking experiences. Partners offered emotional and practical support |
| Lua et al., 2017 | USA | Quantitative | Cross-sectional study | Women diagnosed with vulvodynia (N= 12,584) | To identify the most   common approaches for managing vulvodynia in the US, and the associated comorbidities  and healthcare costs | N/A | Truven Health Analytics MarketScan Commercial Claims and Encounter | Variation in the current management of vulvodynia reflecting the poor current understanding of the etiology and pathophysiology of vulvodynia |
| Mitchell et al., 2021 | Australia | Quantitative | Cross-sectional | Women diagnosed with vulvodynia (N=50) | To explore which healthcare professionals Australian women with vulvodynia seek treatment from, and which treatments are recommended, provided, or prescribed by these healthcare professionals | N/A | Online survey to acquire information regarding the types and number of healthcare professionals consulted and the treatments they offered | High variability of treatments and clinicians seen for vulvodynia. Offered treatments have very little peer-reviewed evidence of effectiveness in vulvodynia |
| Shallcross et al., 2019 | Northwest of England | Qualitative | Cross-sectional | Women diagnosed with vulvodynia (N=8) | To explore the experiences of women diagnosed with vulvodynia within the UK healthcare system, specifically their journey toward diagnosis | N/A | Semi-structured interviews with participants | Women perceived a dismissive healthcare system with an inadequate knowledge of vulvodynia    affecting their psychological wellbeing |
| Templeman et al., 2023 | UK | Qualitative | Cross-sectional | Women diagnosed with vulvodynia (N=6) | To explore the health care experiences of women living with vulvodynia in the UK | N/A | Semi-structured interviews with participants | Many reported feeling their pain was dismissed due to gender, emphasizing that pain management should be prioritized over well-being and mental health |
| Törnävä et al., 2018a | Finland | Quantitative | Quasi-experimental | Student healthcare providers (N=191) | To assess awareness and knowledge of vulvodynia and its care among student healthcare providers before and after Web-based education | Web based education content on vulvodynia and its care | A survey instrument ‘Awareness and knowledge of vulvodynia and its care’ and a web-based questionnaire. | Improved awareness and knowledge of vulvodynia and its care was following web-based education |
| Törnävä et al., 2018b | Finland | Quantitative | Cross-sectional | Healthcare professionals in the student healthcare field (public health nurses)  (N=191) | To investigate awareness and knowledge about vulvodynia and its care among staff in the student healthcare sector in Finland | A web-based questionnaire consisted of individual background factors and the Web based education content on vulvodynia and its care (Törnävä et al., 2018a) | 'Awareness and knowledge of vulvodynia and its care' survey | Knowledge of vulvodynia and its care was estimated to be poor |
| Vicente-Neira et al., 2022 | Spain | Quantitative | Descriptive | Websites on vulvodynia (N=91) | To evaluate the content, quality, and readability of websites containing information on dyspareunia, vaginismus, and vulvodynia in Spanish to inform healthcare professionals | N/A | The DISCERN questionnaire to evaluate the quality of written health information, the Bermúdez-Tamayo questionnaire to evaluate Spanish websites, and the INFLESZ scale to qualify readability | The quality of information was very low and moderate overall quality. Sites on vaginismus and vulvodynia were difficult to read but better readability for websites on dyspareunia |
| Webber et al., 2023 | Canada | Qualitative | Case study | Primary care providers (Physicians and nurses) (N=16) | To provide a better understanding of how rurality impacts the knowledge, diagnosis, and management of vulvodynia by primary care providers practicing in the geographically disparate province of Newfoundland and Labrador, Canada | N/A | Questionnaires assessing the perspectives on barriers to diagnosis and treatment for vulvodynia and semi-structured interviews with participants | Rurality exacerbates concerns in the identification and management of vulvodynia as most underestimated the likelihood to see a patient with vulvodynia in their practice |
